# Fully inverse adsorption enables one-step high-purity C_2_H_2_ separation from ternary C2 mixtures in a robust porous crystal

**DOI:** 10.1038/s41467-025-65057-8

**Published:** 2025-11-18

**Authors:** Mingxing Zhang, Jingui Duan, Yanfei Feng, Junfeng Bai

**Affiliations:** 1 College of Chemistry and Chemical Engineering, Nantong University, Nantong, Jiangsu China; 2 State Key Laboratory of Materials-Oriented Chemical Engineering & School of Chemistry and Molecular Engineering, Nanjing Tech University, Nanjing, China; 3 State Key Laboratory of Chemistry and Utilization of Carbon-Based Energy Resources, College of Chemistry, Xinjiang University, Urumqi, China; 4 Nanjing Tech University Suzhou Future Membrane Technology Innovation Center, Suzhou, China

**Keywords:** Metal-organic frameworks, Coordination chemistry

## Abstract

Direct harvesting of electronic-grade acetylene (C_2_H_2_) from ternary C2 mixtures is a great challenge due to the ubiquitous adsorption preference of conventional porous materials (C_2_H_2_ > ethylene (C_2_H_4_) > ethane (C_2_H_6_)). Here, we report a strategy to reverse this selectivity by leveraging ligand functionalization in porous crystals. Through the incorporation of trifluoromethyl/methyl groups into a pyrazole-carboxylate linker, we engineer a series of MOF-5 analogs. The optimal material, **NTU-98**, fully reverses the adsorption trend of C2 hydrocarbons (C_2_H_6_ > C_2_H_4_ > C_2_H_2_), enabling direct production of C_2_H_2_ with >99.99% purity from ternary feeds at room temperature in one-step. Combined density functional theory calculations and gas-loaded crystallographic analyses unveil the molecular mechanism: methyl groups precisely positioned within the cages enhance host-guest interactions with C_2_H_4_ and C_2_H_6_, while suppressing the binding affinity for C_2_H_2_. This work presents a porous crystal for direct C_2_H_2_ purification from ternary feeds and a blueprint for designing microporous environments targeting challenging separations.

## Abstract (web summary)

In this study, authors report the porous crystal NTU-98, which fully inverts the adsorption sequence of C2 hydrocarbons, thereby enabling the one-step production of ultrahigh-purity acetylene from C2 ternary mixtures under ambient conditions.

## Introduction

Acetylene (C_2_H_2_) is a crucial feedstock in the chemical and semiconductor industries, where ultrahigh-purity electronic-grade C_2_H_2_ is indispensable for manufacturing carbon masks used in photolithography during advanced integrated circuit production^1^. However, industrial C_2_H_2_ streams mainly derived from cracked gas or ethane (C_2_H_6_) dehydrogenation typically contain ternary C2 hydrocarbon mixtures (C_2_H_2,_ C_2_H_4_ and C_2_H_6_) with nearly identical physicochemical properties, making their separation particularly challenging^2,3^. Current purification strategies predominantly rely on solvent extraction, wherein C_2_H_2_ is selectively dissolved into organic solvents and subsequently recovered via desorption^4^. While effective, this process is constrained by the steep temperature dependence of hydrocarbon solubility, necessitating energy-intensive sub-ambient conditions (e.g., −40 °C) to maximize efficiency. Furthermore, solvent regeneration generates substantial liquid waste, and the purity of recovered C_2_H_2_ often falls short of the stringent electronic-grade thresholds in a single operational cycle. These limitations underscore an urgent demand for innovative separation technologies that simultaneously prioritize energy.

Adsorptive separation presents a compelling alternative to conventional absorption methods, particularly for applications requiring high selectivity, energy efficiency, facile regeneration, and operational versatility. Unlike solvent-dependent absorption, adsorption leverages the selective interactions between target molecules and tailored porous adsorbents, thereby enabling precise capture of trace impurities and adaptability to environmentally sensitive processes^5,6^. Microporous materials such as zeolites and activated carbon have been considered as prominent candidates for C_2_H_2_ purification^7,8^. Yet their practical utility is hampered by inherent limitations: elevated temperature for adsorbent regeneration and/or insufficient selectivity. Particularly, the high reactivity of C_2_H_2_ at elevated temperatures may produce undesirable side reactions (e.g., polymerization or decomposition), adding new impurities or blocking the pore of the adsorbent. These challenges persist even in state-of-the-art systems, restricting scalability and industrial adoption. Highlight the need for developing adsorbents with engineered pore geometries and optimized binding affinities—innovations critical to achieving highly pure C_2_H_2_.

Porous coordination polymers (PCPs), also known as metal-organic frameworks (MOFs), have emerged as a versatile platform for light hydrocarbon separations, owing to their precisely engineered pore geometries^9–11^ and chemical functionalities^12–14^ enabled by crystal engineering and isoreticular design principles^15–19^. The high quadrupole moment and π-electron density of C2 gases hydrocarbons drive strong interactions with polar binding sites or open metal centers in PCPs^20^, leading to a typical adsorption sequence of C_2_H_2_ > C_2_H_4_ > C_2_H_6_^21^. However, to facilitate efficient and direct harvesting of highly pure C_2_H_4_ in the adsorption process, strategies involving supramolecular sites and structural dynamics have been developed for tuning the adsorption sequence^22–28^, with notable success in C_2_H_4_ purification via partially reversed selectivity adsorption order (C_2_H_2_ > C_2_H_6_ > C_2_H_4_)^29–31^. However, due to its high quadrupole moment and small kinetic diameter, C_2_H_2_ remains the most strongly adsorbed species in most porous systems. By utilizing a perfluorinated narrow channel, a reversed adsorption order of C2 hydrocarbons has been observed in Zn-FBA; however, the negligible uptake differences between C_2_H_6_-C_2_H_2_ and C_2_H_4_-C_2_H_2_ render C_2_H_2_ purification infeasible from C2 ternary mixtures^32^. In other words, neither a function-driven approach nor a finely tuned structural design of PCPs has successfully achieved electronic-grade C_2_H_2_ purification from ternary C2 mixtures in one-step under industrially relevant conditions.

**Fig. 1:**
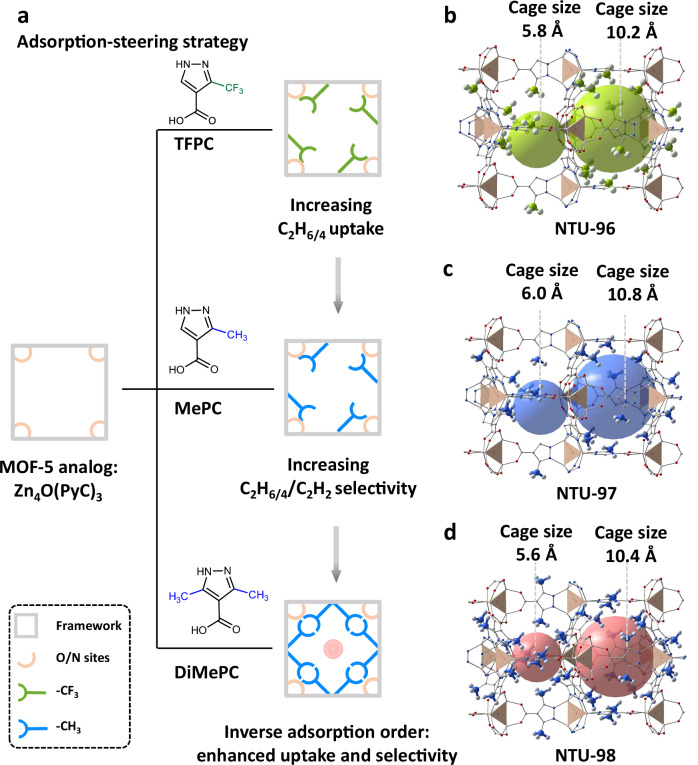
Adsorption-steering strategy in the highly porous Zn_4_O(PyC)_3_ platform. **a** Systematic implantation of inert groups with varying types and loading amounts for enhanced C_2_H_6/4_ uptakes and C_2_H_6/4_/C_2_H_2_ selectivities. **b**–**d** Corresponding structures: **NTU-96,** **NTU-97** and **NTU-98**, respectively. Color code: Zn_4_O, brown tetrahedra; C, gray; O, red; N, blue; H, white; F, cyan; void space, highlighted in green, blue and light red; gray row, fine-tuned structures.

In this work, we address the challenge of purifying C_2_H_2_ from ternary C2 mixtures by adopting a counterintuitive strategy: weakening the host-guest interactions with C_2_H_2_ while enhancing the binding with its saturated counterparts, C_2_H_6_ and C_2_H_4_. To achieve this, we developed an adsorption-steering methodology utilizing a MOF-5 analog platform (Zn_4_O(PyC)_3_, PyC = 4-pyrazolecarboxylate) (Fig. 1a)^33,34^. Through incorporation of inert functional groups (trifluoromethyl and methyl groups) to the PyC ligand, we synthesized a series of MOF-5 analogs and realized several synergistic objectives: (1) maintaining a high surface area (>1000 m^2^ g^−1^) to ensure adequate adsorption capacity; (2) strengthening van der Waals interactions with hydrogen-rich C_2_H_6_ and C_2_H_4_ while simultaneously suppressing the affinity for C_2_H_2_; and (3) enhancing the water-stability of the framework through steric protection of the metal nodes by hydrophobic methyl groups. Of them, **NTU-98**, featuring two methyl groups on the PyC ligand, represents a robust framework and exhibits a fully inversed adsorption order (C_2_H_6_ > C_2_H_4_ > C_2_H_2_), enabling the direct harvesting of high-purity C_2_H_2_ (>99.99%) from C2 ternary mixtures at 298 K. Density functional theory calculations and gas-loaded crystallographic analyses reveal that in **NTU-98**, the abundant methyl groups within the small cage, along with the methyl-decorated nitrogen/oxygen adsorption corners in the large cage, provide stronger interactions with the more saturated C_2_H_6_ and C_2_H_4_, while concurrently diminishing the affinity for C_2_H_2_. This behavior contrasts with other analogs with single methyl or trifluoromethyl group on the ligand, which exhibit significantly limited separation efficiency.

## Results

### Structure and adsorption properties

Colorless transparent crystals of **NTU-96,** **NTU-97** and **NTU-98** were obtained through solvothermal reactions of zinc nitrate hexahydrate and 4-PyC derivatives (TFPC, MePC and DiMePC, respectively) (Fig. 1b–d). Single crystal X-ray diffraction results demonstrated that **NTU-96** and **NTU-97** both crystallize in space group *Fm-3m*, while **NTU-98** crystallizes in *F-43m* (Supplementary Table 1). The similar coordination properties of pyrazolate and carboxylate make these structures analogous to that of MOF-5, featuring a classical node of Zn_4_O (Supplementary Figs. 1–5). But, differently, the coordination of the two N sites of 4-PyC derivatives greatly constraints the rotational freedom of the ligand, yielding the three-dimensional framework with two different cages in **NTU-96** to **NTU-98**. Upon ligand functionalization with trifluoromethyl, methyl, and two methyl groups, the internal diameter of the smaller cubic cage slightly contracts, shrinking from the original 8.0 Å (in Zn_4_O(PyC)_3_) to 5.8, 6.0, and 5.6 Å, respectively (Fig. 1b–d, Supplementary Fig. 6). Note that, although Zn_4_(μ_4_-O)(DiMePC)_3_ was previously investigated for the capture of nerve agents and mustard gas^35^, the reported structure is not exactly the same as that of **NTU-98** (with different crystal symmetries), and the functional implications of the two methyl groups in modulating host–guest interactions remain unexplored. Phase purity of **NTU-96,** **NTU-97** and **NTU-98** were confirmed by the powder X-ray diffraction (PXRD) analysis (Supplementary Figs. 7–9). Thermogravimetric analysis (TGA) of as-synthesized and activated samples confirm that these materials are thermally stable up to 400 °C (Supplementary Figs. 10–15).

N_2_ (77 K) and CO_2_ (195 K) adsorption isotherms were collected to evaluate the permanent microporosity of **NTU-96** to **NTU-98**, respectively. Type-I adsorption isotherms were observed without any hysteresis (Supplementary Figs. 16, 17). **NTU-96** and **NTU-97** exhibit high N_2_ uptake, up to 250.6 and 320.3 cm^3 ^g^−1^, respectively. However, the N_2_ uptake of **NTU-98** (44.5 cm^3 ^g^−1^) is much lower than that of the other two, but it has comparable CO_2_ uptake (240.3 cm^3 ^g^−1^). This is likely due to the blocking effect of aggregated N_2_ at very low temperature. Based on the CO_2_ isotherms, Brunauer-Emmet-Teller (BET) surface areas of the three were calculated to be ∼1150, 1470 and 1245 m^2^ g^−1^, respectively. The derived pore size distribution centers at 8.8 Å in **NTU-96**, 9.6 Å in **NTU-97** and 8.4 Å in **NTU-98**, which are all comparable to the geometrically determined values of the cavities based on the crystal structures.

**Fig. 2:**
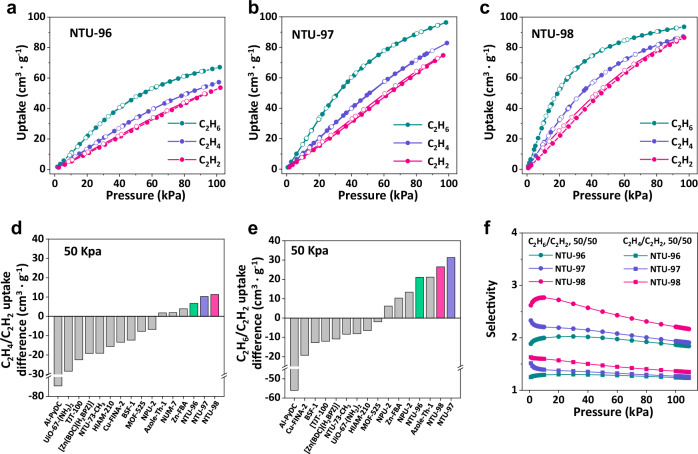
Gas adsorption properties. C_2_H_2_, C_2_H_4_ and C_2_H_6_ adsorption isotherms of **a NTU-96**, **b NTU-97** and **c NTU-98** at 298 K, respectively. Comparison of **d** C_2_H_4_/C_2_H_2_ and **e** C_2_H_6_/C_2_H_2_ uptake difference of the benchmark PCPs at 298 K, 50 kPa. Values for literature materials are taken from refs: Al-PyDC^43^, Uio-67-(NH_2_)_2_^31^, TJT-100^29^, [Zn(BDC)(H_2_BPZ)].4H_2_O^44^, NTU-73-CH_3_^45^, HIAM-210^46^, Cu-FINA-2^47^, BSF-1^48^, MOF-525^49^, NPU-2^30^, Azole-Th-1^25^ and NUM-7^50^. **f** IAST selectivity of C_2_H_6_/C_2_H_2_ (1/1, v/v) and C_2_H_4_/C_2_H_2_ (1/1, v/v) at 298 K.

To evaluate the role of nanopores in PCPs, single-component adsorption isotherms of C_2_H_2_, C_2_H_4_, and C_2_H_6_ were collected at 273–308 K, along with data for the precursor Zn_4_O(PyC)_3_ (Fig. 2, Supplementary Figs. 18–53, and Supplementary Table 2). Zn_4_O(PyC)_3_ exhibits a preference for C_2_H_6_ adsorption, with nearly overlapping adsorption isotherms for C_2_H_2_ and C_2_H_4_. This observation correlates with the zero coverage isosteric heat of adsorption (*Q*_st_) values (C_2_H_6_: 25 kJ mol^−1^; C_2_H_4_: 22.6 kJ mol^−1^ and C_2_H_2_: 23.8 kJ mol⁻¹). In contrast, **NTU-96** demonstrates a completely reversed adsorption order for these gases. The *Q*_st_ values for **NTU-96** are significantly higher, measuring 29.4 kJ mol^−1^ for C_2_H_6_, 27.7 kJ mol^−1^ for C_2_H_4_, and 26.5 kJ mol^−1^ for C_2_H_2_, all exceeding those of Zn_4_O(PyC)_3_ (Table 1 and Supplementary Figs. 54–66). The elevated *Q*_st_ for C_2_H_2_ suggests that the polar trifluoromethyl groups have a synergistic effect with some of the original adsorption sites rather than interfering with or shielding the adsorption sites. However, the uptake difference between C_2_H_4_ and C_2_H_2_ remains relatively modest, at 6.7 cm^3 ^g^−1^ at 298 K and 50 kPa. Upon substituting the trifluoromethyl with methyl group, **NTU-97** also exhibits a completely reversed adsorption order for C2 hydrocarbons. This alteration is accompanied by a moderate increase in the uptake difference between C_2_H_4_ and C_2_H_2_, recorded at 10.3 cm^3 ^g^−1^, despite a significantly larger uptake difference of 31.3 cm^3 ^g^−1^ between C_2_H_6_ and C_2_H_2_. Importantly, **NTU-98**, which contains two implanted methyl groups, demonstrates not only a fully inversed adsorption order, but also steeper uptake profiles for all three gases (Fig. 2a–c). When compared to benchmark materials, **NTU-98** exhibits the largest uptake difference for C_2_H_4_ and C_2_H_2_ (11.3 cm^3 ^g^−1^), while its uptake difference for C_2_H_6_ and C_2_H_2_ (26.5 cm^3 ^g^−1^) is only slightly smaller than that of **NTU-97** (31.3 cm^3 ^g^−1^) (Fig. 2d, e and Supplementary Table 3). Additionally, the *Q*_st_ values for **NTU-98** are 32.2 kJ mol^−1^ for C_2_H_6_ and 27.3 kJ mol^−1^ for C_2_H_4_, both of which are marginally higher than those for **NTU-97** (28 and 25 kJ mol^−1^, respectively). However, these values are still lower than that of Zn-FBA (42.8 and 39.8 kJ mol^−1^), indicating a reduced energy requirement during cyclic operations. Considering the emerging adsorption order and systematically tuned uptake differences, ideal adsorbed solution theory (IAST)^36–38^ was applied to calculate the adsorption selectivity (Fig. 2f, Supplementary Figs. 67–71). **NTU-98** demonstrated exceptional adsorption selectivity for equimolar C_2_H_4_/C_2_H_2_ and C_2_H_6_/C_2_H_2_ mixtures, achieving values of 1.62 and 2.62, respectively, at 298 K and low pressure. These selectivities further increased at 273 K, reaching 1.95 (C_2_H_4_/C_2_H_2_) and 3.98 (C_2_H_6_/C_2_H_2_), underscoring its temperature-dependent performance. In contrast, **NTU-96** (1.25 and 1.88), **NTU-97** (1.51 and 2.32), and Zn_4_O(PyC)_3_ (0.93 and 1.09) exhibited markedly lower selectivities under identical conditions. The results highlight **NTU-98** as a superior adsorbent for the challenging separation of C_2_H_2_ from C_2_H_6_ and C_2_H_4_-containing mixtures.

**Table 1:** Summarized surface areas, uptakes, adsorption heats and selectivities of the PCPs

PCPs	BET(m^2^ g^−1^)	Uptake (cm^3^ g^−1^)50 kPa, 298 K			*Q*_st_ (kJ mol^−1^)				Selectivity (v/v = 1:1)298 K		
		C_2_H_2_	C_2_H_4_	C_2_H_6_	C_2_H_2_		C_2_H_4_	C_2_H_6_	C_2_H_4_/C_2_H_2_		C_2_H_6_/C_2_H_2_
Zn_4_O(PyC)_3_	1350	39.9	40.6	61.5	23.8		22.6	25.1	0.93		1.09
**NTU-96**	1150	26.7	33.4	47.8	26.5		27.1	29.0	1.25		1.88
**NTU-97**	1470	38.5	48.8	69.8	23.3		25.0	28.0	1.51		2.32
**NTU-98**	1245	53.5	64.8	80.0	23.5		27.3	32.2	1.62		2.62

### Adsorption mechanism studies

**Fig. 3:**
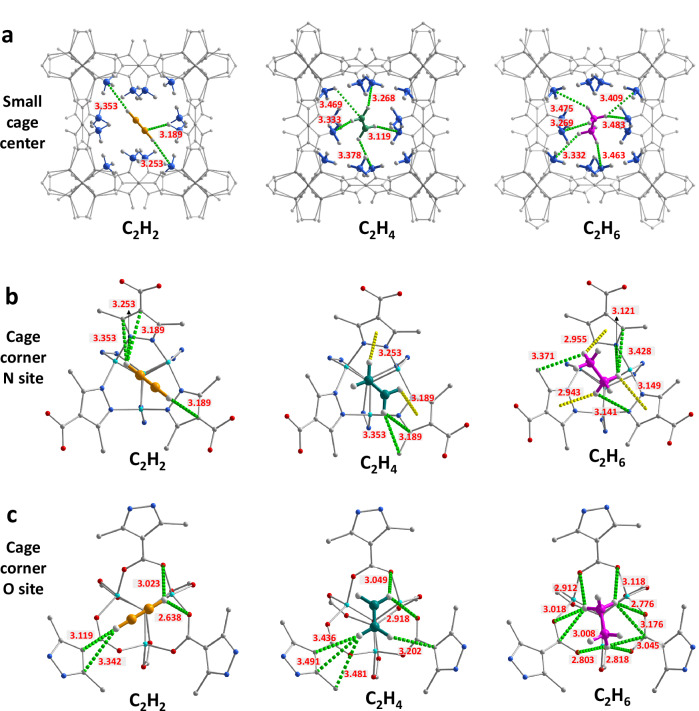
DFT-D calculated host-guest interactions of NTU-98. **a** C2 hydrocarbons located at the small cage center, **b** at the cage corner N site, and **c** at the cage corner O site. Color code: Zn, light green; C, gray; O, red; N, blue; H, white; C_2_H_2_: orange; C_2_H_4_: dark green; C_2_H_6_, purple; The green and yellow dashed lines indicate hydrogen bonding and C-H…**π** interaction, respectively.

To elucidate the molecular interactions between **NTU-98** and C2 hydrocarbons, we conducted dispersion-corrected density functional theory (DFT-D) calculations. Our analysis reveals that the primary adsorption sites for all three C2 hydrocarbons are localized at the center of the small cage and the corners of the large cage within the **NTU-98** framework. In Fig. 3, we show schematically the DFT-D-optimized adsorption structures of the three gas molecules at various adsorption sites. Within the small cage, the two H_C2H2_ interact with two C_CH3_ from DiMePc, forming C–H···C interactions with distances ranging from 3.253 to 3.353 Å (Fig. 3a). Additionally, a hydrogen bond is established between a H_CH3_ and a C_C2H2_ at a distance of 3.189 Å. Interestingly, four hydrogen bonds are observed between H_C2H4_ and C_CH3_ from DiMePc, with bond distances varying from 3.119 to 3.378 Å, coupled with a hydrogen bond between H_CH3_ and C_C2H4_ at 3.469 Å. In addition, due to the increased H^δ⁺^ on C_2_H_6_, six hydrogen bonds are formed with the C_CH3_ groups, with bond lengths ranging from 3.269 to 3.483 Å. The increased number of hydrogen bonds, although each has slightly different bond length, clearly indicates progressively stronger host–guest interactions for C_2_H_4_ and especially C_2_H_6_. Owing to the asymmetric nature of the ligand, the metal nodes within the framework can exhibit mixed coordination environments, featuring both Zn–N and Zn–O bonds. To accurately model the host-guest interactions, we defined two representative configurations for the large cage corners, differentiated by the identity of the coordinating atom (either nitrogen or oxygen). In both cases, C_2_H_6_ and C_2_H_4_ form more hydrogen bonds and C–H···π interactions with DiMePc than C_2_H_2_ (Fig. 3b, c). For comparison, we also performed calculations on the C2 hydrocarbon adsorption in the parent framework Zn_4_O(PyC)_3_. In the absence of functional methyl groups, adsorption primarily occurs at (i) the window aperture bridging small and large cages, and (ii) the N/O-defined adsorption corners. Meanwhile, the number of hydrogen bonds at N/O corners increases with the number of hydrogen atoms on the guest molecule (C_2_H_6_ > C_2_H_4_ > C_2_H_2_). However, at the window aperture, all three C2 species form nearly the same number of hydrogen bonds (C_2_H_2_: 8, C_2_H_4_: 7 and C_2_H_6_: 8), suggesting non-discriminative binding (Supplementary Fig. 72). This contrast—between site-specific selectivity and aperture-driven uniformity—explains the diminished C2 selectivity of the parent framework. These observations are further evident from the calculated static gas binding energies at various adsorption sites (Table 2), which are also fully consistent with the experimental *Q*_st_ values.

**Table 2:** DFT-D calculated static binding energies at various adsorptions sites (kJ mol^−1^)

	Cage corner O site			Cage corner N site			Cage window			Small cage center		
	C_2_H_2_	C_2_H_4_	C_2_H_6_	C_2_H_2_	C_2_H_4_	C_2_H_6_	C_2_H_2_	C_2_H_4_	C_2_H_6_	C_2_H_2_	C_2_H_4_	C_2_H_6_
Zn_4_O(PyC)_3_	22.4	24.3	24.5	21.0	22.7	23.6	25.4	22.7	24.2			
**NTU-98**	23.3	25.8	27.4	23.6	26.4	28.0				24.5	27.5	30.7

To validate the computational modeling results, we further experimentally determined the gas-loaded crystal structures by single-crystal X-ray diffraction. Due to the high crystal symmetry, accurately resolving the orientations of the ligand and guest gas molecules is challenging, resulting in apparent orientational disorder in the experimental structures. Nevertheless, the experimental results confirm that the three primary adsorption sites—the center of small cage and the corners of large cage featuring N or O atoms—closely match the predictions from the modeling. Consistent with the calculations, gas molecules with higher H^δ+^ content exhibit stronger interactions (Supplementary Fig. 73), and the experimentally observed hydrogen bond lengths are in close agreement with the calculated values. The adsorption kinetics are of the same order of magnitude, indicating a limited influence on the overall selectivity (Supplementary Fig. 73). Therefore, the high selectivity of **NTU-98** for C_2_H_4_ and C_2_H_6_ over C_2_H_2_ arises from the synergistic effect of strategically positioned methyl groups within both cages and the N/O-decorated adsorption sites. These features collectively enhance the cooperative interactions with the more saturated C_2_H_4_/C_2_H_6_, while simultaneously diminishing the affinity toward the more polarizable C_2_H_2_. This molecular-level design leverages intrinsic differences in C–H bond acidity across the C2 hydrocarbon series.

### Dynamic breakthrough experiments

**Fig. 4:**
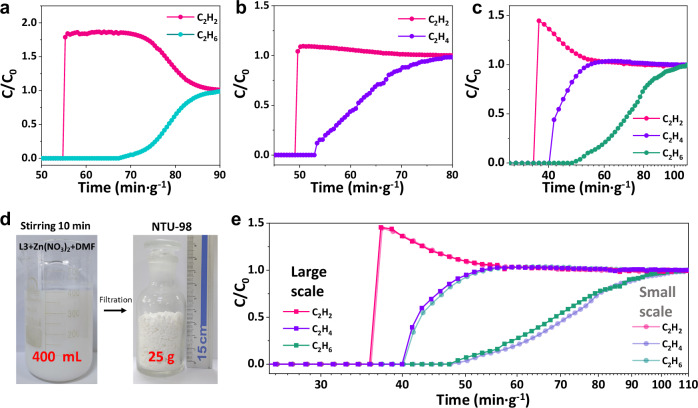
Breakthrough experiments. Breakthrough curves of **NTU-98** for **a** C_2_H_6_/C_2_H_2_ (1/1, v/v, 0.9 mL·min^−1^), **b** C_2_H_4_/C_2_H_2_ (1/1, v/v, 0.9 mL·min^−1^), **c** C_2_H_2_/C_2_H_4_/C_2_H_6_ (90/9/1,v/v/v, 2 mL·min^−1^). **d** Photos of the large-scale synthesis for **NTU-98** (L3 = DiMePC ligand; DMF =  Dimethylformamide). **e** Comparisons of breakthrough curves of small-scale and large-scale synthesized **NTU-98** for C_2_H_2_/C_2_H_4_/C_2_H_6_ (90/9/1, v/v/v, 2 mL·min^−1^) mixtures. The temperature and pressure for all breakthrough experiments is 298 K and 1 bar.

Considering the distinct adsorption order, breakthrough experiments were carried out on the three PCPs. After initial activation, the three samples were loaded into a column, followed by further activation. The system was flushed with He until no other signal was detected. Then the corresponding feed gas was introduced into the packed column. The clear time intervals indicate that **NTU-98** can effectively separate the binary mixtures of C_2_H_6_/C_2_H_2_ (v/v, 1/1) and C_2_H_4_/C_2_H_2_ (v/v, 1/1) (Fig. 4a, b and Supplementary Figs. 75, 76). After changing the feed gas to the C2 ternary mixtures, C_2_H_2_ eluted first out of the fixed bed of **NTU-98** at 56.5 min·g^−1^, and the C_2_H_4_ breakthrough the column at 57.6 min·g^−1^, followed by C_2_H_6_ at 67.1 min·g^−1^, well matching the inversed adsorption behavior of **NTU-98**. With reference to the higher ratio of C_2_H_2_ during the deep purification step, the feed gas ratio was then change to 90/9/1 (C_2_H_2_/C_2_H_4_/C_2_H_6_: v/v/v)^39,40^. Consistently, same elution sequences were observed, of which C_2_H_2_ with high purity (>99.99%) was detected at about 37.2 min·g^−1^, followed by C_2_H_4_ at 41.6 min·g^−1^, and C_2_H_6_ at 48.8 min·g^−1^, the corresponding C_2_H_2_ yield is 8.64 mL g^−^¹ (Fig. 4c). Despite extensive research in PCP chemistry, the direct one-step purification of C_2_H_2_ with high purity from ternary C2 mixtures remains, to the best of our knowledge, unreported. In comparison, **NTU-96** can also separate these ternary mixtures, but the separation time for harvesting of electronic grade C_2_H_2_ is short (Supplementary Figs. 77, 78).

For practical gas separation applications, scale-up synthesis and water stability of the adsorbent material are also two important factors to consider^41^. We found that rapid, scaled up synthesis of **NTU-98** can be readily achieved. By adding certain amount of NH_3_·H_2_O into the stirring solution (400 mL scale) of the corresponding reactants, ∼25 g of **NTU-98** can be obtained in 10 min, making the space-time yield as high as about 9000 kg/m^3^/day (Fig. 4d). Phase purity and porosity of the large-scale synthesized **NTU-98** were validated by PXRD and gas adsorption isotherms (Supplementary Fig. 79). Importantly, large-scale synthesized **NTU-98** demonstrates nearly the same separation performance for C2 hydrocarbons (Fig. 4e). Furthermore, we found that **NTU-98** exhibits excellent chemical stability. Even after immersion in solutions with pH ranging from 2 to 12 for one week, the sample largely retained its crystallinity and gas uptake capacity (Supplementary Fig. 80). Such high-water stability is fully expected because the two methyl groups on each ligand effectively shield the Zn-O coordination bonds from hydrolysis. Thanks to its stability, **NTU-98** does not suffer any notable performance loss during cycling breakthrough experiments (Supplementary Fig. 81).

## Discussion

Addressing the challenge of directly harvesting high-purity C_2_H_2_ from ternary C2 mixtures, we present an adsorption-steering strategy that optimizes ensemble host–guest interactions in a family of porous crystals. NTU-98, featuring dual methyl groups positioned at adsorption corners, exhibits a fully inverted adsorption hierarchy (C_2_H_6_ > C_2_H_4_ > C_2_H_2_). Gas-loaded crystallography and DFT-D analyses reveal that the synergistic interplay between sterically placed methyl groups and N/O-functionalized nodes enhances binding toward saturated hydrocarbons while diminishing affinity for polarizable C₂H₂. Of them, NTU-98 combines high structural robustness and synthetic scalability, enabling direct production of electronic-grade C_2_H_2_ (>99.99%) from ternary C2 feeds in one step under ambient conditions, which has not been demonstrated in earlier PCP studies. This design strategy is broadly applicable to other porous frameworks, providing a framework for exploring inverse adsorption hierarchies and advancing adsorbents for challenging separations.

## Methods

### Synthesis of NTU-96

5-(trifluoromethyl)-1H-pyrazole-4-carboxylic acid (TFPC) (4 mg, 0.022 mmol) and Zn(NO_3_)2•6H_2_O (12 mg, 0.04 mmol) were added to the 1 mL mixed solvent of DMA/H_2_O (v/v, 1/1), sonicated and placed into a 10 mL glass bottle, and heated in an oven at 120 °C for 48 h. After the end of the reaction, colorless crystals are obtained. The NTU-96 crystals are washed with DMA and stored dry at room temperature.

### Synthesis of NTU-97

3-methyl-1H-pyrazole-4-carboxylic acid (MePC) (4 mg, 0.032 mmol) and Zn(NO_3_)2•6H_2_O (20 mg, 0.07 mmol) were added to the 1 mL mixed solvent of DEF/H_2_O (v/v, 3/1), sonicated, and transferred to a 10 mL glass bottle. Subsequently, the glass bottles were heated in an oven at 85 °C for 48 h. After the end of the reaction, colorless crystals were obtained. The NTU-97 crystals are washed with DEF and stored dry at room temperature.

### Synthesis of NTU-98 (small-scale)

3,5-Dimethyl-1H-pyrazole-4-carboxylic acid (DiMePC, 14 mg, 0.1 mmol) and zinc nitrate hexahydrate (Zn(NO₃)₂·6H₂O, 27 mg, 0.09 mmol) were dissolved in 1 mL of N,N’-dimethylformamide (DMF) with the aid of sonication. The solution was then transferred into a 10 mL glass vial and heated at 120 °C for 24 h. Upon cooling to room temperature, colorless crystals of NTU-98 were obtained. The crystals were washed with fresh DMF and dried under ambient conditions.

### Synthesis of NTU-98 (large-scale)

For scale-up, the corresponding reactants (DiMePC: 14 g, 0.1 mol and Zn(NO₃)₂·6H₂O: 27 g, 0.09 mol) were dissolved in 400 mL of DMF with stirring, followed by the addition of an appropriate amount of aqueous ammonia (NH_3_·H_2_O). Crystallization occurred rapidly, yielding ~25 g of NTU-98 within 10 min.

### Gas adsorption measurements

Single gas adsorption isotherms were performed on a Belsorp volumetric adsorption instrument (BEL Japan Corp.). In the sorption measurements, ultra-high-purity grade gases of C_2_H_2_, C_2_H_4_, C_2_H_6_ and CO_2_ were used throughout the adsorption experiments.

### Dynamic breakthrough experiments

Breakthrough experiments were performed on the Beifang Gaorui CT-4 system. The initial activated samples were tightly packed into a stainless-steel column (φ = 0.40 cm, L = 30 cm). Then, the column was activated under vacuum at the corresponding temperature and then swept with helium (He) flow to remove impurities. Until no signal was detected, the gas flow was dosed into the column. Breakpoints were determined by gas chromatography. Between cycling experiments, re-generation can be achieved under high vacuum at 393 K for half-hour. Pressure of the feed gas is 1 bar. For breakthrough experiments, the mixtures of C_2_H_6_/C_2_H_2_, C_2_H_4_/C_2_H_2_ and C_2_H_6_/C_2_H_4_/C_2_H_2_ were obtained by utilizing a premix gas cylinder.

## Supplementary information

Supplementary Information

Transparent Peer Review file

## Source data

Source Data

## Data Availability

The crystal data generated in this study have been deposited in the Cambridge Crystallographic Data Centre (CCDC) under accession code 2434597-2434601 and 2434605 [https://www.ccdc.cam.ac.uk/structures]. Source data of the sorption tests, gas adsorption enthalpies, selectivities and break through tests that support the findings of this study are provided as a SourceData file (ref. ^42^). Source data are provided with this paper.

## References

[1] Vininski, P. J. V. Acetylene process gas purification methods and systems. US patent US8182589B2 (2009).

[2] PASSLER, P. Acetylene In *Ullmann’s**Encyclopedia of Industrial Chemistry* (Wiley, 2011).

[3] Adil, K. et al. Gas/vapour separation using ultra-microporous metal–organic frameworks: insights into the structure/separation relationship. *Chem. Soc. Rev.* **46**, 3402–3430 (2017). 28555216 10.1039/c7cs00153c

[4] Weissermel, K. & Arpe, H.-J. Acetylene. In *Industrial Organic Chemistry* (Wiley, 2003).

[5] Yang, S.-Q., Hu, T.-L. & Chen, B. Microporous metal-organic framework materials for efficient capture and separation of greenhouse gases. *Sci. China Chem.* **66**, 2181–2203 (2023).

[6] Lan, T. et al. Opportunities and critical factors of porous metal–organic frameworks for industrial light olefins separation. *Mater. Chem. Front.* **4**, 1954–1984 (2020).

[7] Chai, Y. et al. Control of zeolite pore interior for chemoselective alkyne/olefin separations. *Science* **368**, 1002–1006 (2020). 32467390 10.1126/science.aay8447

[8] Choi, B.-U., Choi, D.-K., Lee, Y.-W., Lee, B.-K. & Kim, S.-H. Adsorption equilibria of methane, ethane, ethylene, nitrogen, and hydrogen onto activated carbon. *J. Chem. Eng. Data* **48**, 603–607 (2003).

[9] Skrabalak, S. E. & Vaidhyanathan, R. The chemistry of metal organic framework materials. *Chem. Mater.* **35**, 5713–5722 (2023).

[10] Zhai, Q.-G., Bu, X., Zhao, X., Li, D.-S. & Feng, P. Pore space partition in metal–organic frameworks. *Acc. Chem. Res.* **50**, 407–417 (2017). 28106984 10.1021/acs.accounts.6b00526

[11] Tian, Y. & Zhu, G. Porous aromatic frameworks (PAFs). *Chem. Rev.* **120**, 8934–8986 (2020). 32101403 10.1021/acs.chemrev.9b00687

[12] Das, M. C., Xiang, S., Zhang, Z. & Chen, B. Functional mixed metal–organic frameworks with metalloligands. *Angew. Chem. Int. Ed.* **50**, 10510–10520 (2011). 10.1002/anie.201101534 21928461

[13] Kitagawa, S., Kitaura, R. & Noro, S. -i. Functional porous coordination polymers. *Angew. Chem. Int. Ed.* **43**, 2334–2375 (2004). 10.1002/anie.200300610 15114565

[14] Ji, Z., Wang, H., Canossa, S., Wuttke, S. & Yaghi, O. M. Pore chemistry of metal–organic frameworks. *Adv. Funct. Mater.* **30**, 2000238 (2020).

[15] Zhao, X., Wang, Y., Li, D.-S., Bu, X. & Feng, P. Metal–organic frameworks for separation. *Adv. Mater.* **30**, 1705189 (2018). 10.1002/adma.201705189 29582482

[16] Wan, J. et al. Molecular sieving of propyne/propylene by a scalable nanoporous crystal with confined rotational shutters. *Angew. Chem. Int. Ed.* **62**, e202316792 (2023). 10.1002/anie.202316792 37955415

[17] Yang, W. et al. Regulating the dynamics of interpenetrated porous frameworks for inverse C_2_H_6_/C_2_H_4_ separation at elevated temperature. *Angew. Chem. Int. Ed.* **64**, e202425638 (2025). 10.1002/anie.202425638 39992066

[18] Zhang, M. et al. Fine tuning of MOF-505 analogues to reduce low-pressure methane uptake and enhance methane working capacity. *Angew. Chem. Int. Ed.* **56**, 11426–11430 (2017). 10.1002/anie.201704974 28707307

[19] Zheng, B., Bai, J., Duan, J., Wojtas, L. & Zaworotko, M. J. Enhanced CO_2_ binding affinity of a high-uptake rht-type metal−organic framework decorated with acylamide groups. *J. Am. Chem. Soc.* **133**, 748–751 (2011). 21174431 10.1021/ja110042b

[20] Wu, Y. & Weckhuysen, B. M. Separation and purification of hydrocarbons with porous materials. *Angew. Chem. Int. Ed.* **60**, 18930–18949 (2021). 10.1002/anie.202104318 PMC8453698 33784433

[21] Yang, S. et al. Supramolecular binding and separation of hydrocarbons within a functionalized porous metal–organic framework. *Nat. Chem.* **7**, 121–129 (2015). 10.1038/nchem.2114 25615665

[22] Wang, G.-D. et al. Scalable synthesis of robust MOF for challenging ethylene purification and propylene recovery with record productivity. *Angew. Chem. Int. Ed.* **63**, e202319978 (2024). 10.1002/anie.202319978 38369652

[23] Geng, S. et al. Scalable room-temperature synthesis of highly robust ethane-selective metal–organic frameworks for efficient ethylene purification. *J. Am. Chem. Soc.* **143**, 8654–8660 (2021). 34077659 10.1021/jacs.1c02108

[24] Su, K., Wang, W., Du, S., Ji, C. & Yuan, D. Efficient ethylene purification by a robust ethane-trapping porous organic cage. *Nat. Commun.* **12**, 3703 (2021). 34140501 10.1038/s41467-021-24042-7 PMC8211788

[25] Xu, Z. et al. A robust Th-azole framework for highly efficient purification of C_2_H_4_ from a C_2_H_4_/C_2_H_2_/C_2_H_6_ mixture. *Nat. Commun.* **11**, 3163 (2020). 32572030 10.1038/s41467-020-16960-9 PMC7308359

[26] Zhang, P. et al. Ultramicroporous material based parallel and extended paraffin nano-trap for benchmark olefin purification. *Nat. Commun.* **13**, 4928 (2022). 35995798 10.1038/s41467-022-32677-3 PMC9395351

[27] Li, L. et al. Ethane/ethylene separation in a metal-organic framework with iron-peroxo sites. *Science* **362**, 443–446 (2018). 30361370 10.1126/science.aat0586

[28] Dong, Q. et al. Tuning gate-opening of a flexible metal–organic framework for ternary gas sieving separation. *Angew. Chem. Int. Ed.* **59**, 22756–22762 (2020). 10.1002/anie.202011802 32876973

[29] Hao, H.-G. et al. Simultaneous trapping of C_2_H_2_ and C_2_H_6_ from a ternary mixture of C_2_H_2_/C_2_H_4_/C_2_H_6_ in a robust metal–organic framework for the purification of C_2_H_4_. *Angew. Chem. Int. Ed.* **57**, 16067–16071 (2018). 10.1002/anie.201809884 30338921

[30] Zhu, B. et al. Pore engineering for one-step ethylene purification from a three-component hydrocarbon mixture. *J. Am. Chem. Soc.* **143**, 1485–1492 (2021). 33439004 10.1021/jacs.0c11247 PMC8297724

[31] Gu, X.-W. et al. Immobilization of Lewis basic sites into a stable ethane-selective MOF enabling one-step separation of ethylene from a ternary mixture. *J. Am. Chem. Soc.* **144**, 2614–2623 (2022). 35109657 10.1021/jacs.1c10973

[32] Yang, L. et al. Adsorption in reversed Order of C2 hydrocarbons on an ultramicroporous fluorinated metal-organic framework. *Angew. Chem. Int. Ed.* **61**, e202204046 (2022). 10.1002/anie.202204046 35404504

[33] Li, H., Eddaoudi, M., O’Keeffe, M. & Yaghi, O. M. Design and synthesis of an exceptionally stable and highly porous metal-organic framework. *Nature* **402**, 276–279 (1999).

[34] Tu, B. et al. Ordered vacancies and their chemistry in metal–organic frameworks. *J. Am. Chem. Soc.* **136**, 14465–14471 (2014). 25229624 10.1021/ja5063423

[35] Montoro, C. et al. Capture of nerve agents and mustard gas analogues by hydrophobic robust MOF-5 type metal–organic frameworks. *J. Am. Chem. Soc.* **133**, 11888–11891 (2011). 21761835 10.1021/ja2042113

[36] Cessford, N. F., Seaton, N. A. & Düren, T. Evaluation of ideal adsorbed solution theory as a tool for the design of metal–organic framework materials. *Ind. Eng. Chem. Res.* **51**, 4911–4921 (2012).

[37] Bae, Y.-S. et al. Separation of CO_2_ from CH_4_ using mixed-ligand metal−organic frameworks. *Langmuir* **24**, 8592–8598 (2008). 18616225 10.1021/la800555x

[38] Krishna, R. Metrics for evaluation and screening of metal–organic frameworks for applications in mixture separations. *ACS Omega* **5**, 16987–17004 (2020). 32724867 10.1021/acsomega.0c02218 PMC7379136

[39] Yan, B., Cheng, Y. & Jin, Y. Cross-scale modeling and simulation of coal pyrolysis to acetylene in hydrogen plasma reactors. *AlChE J.* **59**, 2119–2133 (2013).

[40] Yan, B., Xu, P., Jin, Y. & Cheng, Y. Understanding coal/hydrocarbons pyrolysis in thermal plasma reactors by thermodynamic analysis. *Chem. Eng. Sci.* **84**, 31–39 (2012).

[41] Yan, B., Xu, P., Guo, C. Y., Jin, Y. & Cheng, Y. Experimental study on coal pyrolysis to acetylene in thermal plasma reactors. *Chem. Eng. J.* **207**, 109–116 (2012).

[42] Zhang, M., Duan, J., Feng, Y. & Bai, J. Fully inverse adsorption enables one-step high-purity C_2_H_2_ separation from ternary C2 mixtures in a robust porous crystal. figshare 10.6084/m9.figshare.29815517 (2025). 10.1038/s41467-025-65057-8 PMC12627629 41253810

[43] Wu, E. et al. Incorporation of multiple supramolecular binding sites into a robust MOF for benchmark one-step ethylene purification. *Nat. Commun.* **14**, 6146 (2023). 37783674 10.1038/s41467-023-41692-x PMC10545795

[44] Wang, G.-D. et al. One-step C_2_H_4_ purification from ternary C_2_H_6_/C_2_H_4_/C_2_H_2_ mixtures by a robust metal–organic framework with customized pore environment. *Angew. Chem. Int. Ed.* **61**, e202205427 (2022). 10.1002/anie.202205427 35499196

[45] Li, Y., Wu, Y., Zhao, J., Duan, J. & Jin, W. Systemic regulation of binding sites in porous coordination polymers for ethylene purification from ternary C2 hydrocarbons. *Chem. Sci.* **15**, 9318–9324 (2024). 38903240 10.1039/d4sc02659d PMC11186340

[46] Liu, J., Wang, H. & Li, J. Pillar-layer Zn–triazolate–dicarboxylate frameworks with a customized pore structure for efficient ethylene purification from ethylene/ethane/acetylene ternary mixtures. *Chem. Sci.* **14**, 5912–5917 (2023). 37293648 10.1039/d3sc01134h PMC10246669

[47] Wu, X.-Q. et al. Understanding how pore surface fluorination influences light hydrocarbon separation in metal–organic frameworks. *Chem. Eng. J.* **407**, 127183 (2021).

[48] Zhang, Y., Yang, L., Wang, L., Duttwyler, S. & Xing, H. A microporous metal-organic framework supramolecularly assembled from a cuii dodecaborate cluster complex for selective gas separation. *Angew. Chem. Int. Ed.* **58**, 8145–8150 (2019). 10.1002/anie.201903600 30974040

[49] Wang, Y. et al. One-step ethylene purification from an acetylene/ethylene/ethane ternary mixture by cyclopentadiene cobalt-functionalized metal–organic frameworks. *Angew. Chem. Int. Ed.* **60**, 11350–11358 (2021). 10.1002/anie.202100782 33661542

[50] Sun, F.-Z. et al. Microporous metal–organic framework with a completely reversed adsorption relationship for C2 hydrocarbons at room temperature. *ACS Appl. Mater. Interfaces* **12**, 6105–6111 (2020). 31922384 10.1021/acsami.9b22410

