# Supplementary material for: Fully inverse adsorption enables one-step high-purity C2H2 separation from ternary C2 mixtures in a robust porous crystal
Source: Nat Commun. 2025 Nov 18;16:10082. doi: 10.1038/s41467-025-65057-8 (PMC12627629; doi:10.1038/s41467-025-65057-8)
Supplement: Supplementary file 1 — Supplementary Information [file 41467_2025_65057_MOESM1_ESM.pdf]

SUPPLEMENTARY INFORMATION

Fully inverse adsorption enables one-step high-purity C_2H_2 separation from ternary C2 mixtures in a robust porous crystal

Mingxing Zhang,[1] Jingui Duan,[2, 3, 4, *] Yanfei Feng[2] and Junfeng Bai[2, *]

Affiliations:

[1] College of Chemistry and Chemical Engineering, Nantong University, Nantong, Jiangsu 226019, China

[2] State Key Laboratory of Materials-Oriented Chemical Engineering, School of Chemistry and Molecular Engineering, Nanjing Tech University, Nanjing 211816, China

[3] State Key Laboratory of Chemistry and Utilization of Carbon-Based Energy Resources, College of Chemistry, Xinjiang University, Urumqi, 830017, China

[4] Nanjing Tech University Suzhou Future Membrane Technology Innovation Center, Suzhou 215300, China.

* Correspondence and requests for materials should be addressed to J.D. (E-mail: duanjingui@njtech.edu.cn) or to J.B. (E-mail: bjunfeng@njtech.edu.cn).

General procedures and materials

All the reagents and solvents were commercially available and were used as received without further purification.

Fourier-transform Infrared (FT-IR) spectra

The FT-IR spectra were recorded from KBr pellets in the range of 4000–600 cm^{-1} on a VECTOR 22 spectrometer.

Thermogravimetric (TG) analyses

TG analyses were performed using a STA 209 F1 (NETZSCH Instruments) thermo-microbalance, heating from room temperature to 600°C at a rate of 10 °C/min under nitrogen flow.

Powder X-Ray Diffraction (PXRD)

Experimental PXRD patterns were measured using Bruker AXS D8 Advance (test conditions: 40 kV, 40 mA, CuKα, λ = 1.5418 Å, scanning range 5-40°). Simulated powder patterns from single-crystal X-ray diffraction data were generated using Mercury 1.4.2 software.

Single Crystal X-Ray Crystallography

Single-crystal X-ray diffraction data was collected on a Bruker Smart Apex CCD diffractometer at 298 K using graphite monochromator Mo Kα radiation (λ = 0.71073 Å). Data reduction was made with the Bruker Saint program. The structure was solved by direct methods and refined with full-matrix least-squares technique using the SHELXTL package [1]. Organic hydrogen atoms were placed in calculated positions with isotropic displacement parameters set to 1.2 × Ueq of the attached atom. For as-synthesized phases, the unit cell includes disordered water molecules, which could not be modeled as discrete atomic sites. We employed PLATON/SQUEEZE[2, 3] to calculate the diffraction contribution of the solvent molecules and, thereby, to produce a set of solvent-free diffraction intensities; structures were then refined again using the data generated. For in-situ measurements, the fully activated **NTU-96 to NTU-98** that loaded inside the half-opened glass tube was purged by C_2H_2, C_2H_4 and C_2H_6 at 298 K with pressure of 1 bar, respectively. The tubes were then sealed under corresponding atmosphere with hot candle. The crystal measurements were performed on a Bruker Smart Apex CCD diffractometer at 298 K. CCDC 2434594-2434605 contains the supplementary crystallographic data for this paper. These data can be obtained free of charge from The Cambridge Crystallographic Data Centre via www.ccdc.cam.ac.uk/data_request/cif. Crystal data are summarized in Table S1.

Sample activation

Solvent-exchanged **NTU-96** and **NTU-98** crystals were prepared by immersing the as-synthesized samples in dry

MeOH for 3 days to remove the nonvolatile solvents, and the extract was decanted every 8 h and fresh MeOH was replaced. The completely activated sample was obtained by heating the solvent-exchanged sample at 25°C for 2 h and then 120°C for 8 h under a dynamic high vacuum.

Solvent-exchanged **NTU-97** were prepared by immersing the as-synthesized samples in dry EtOH for 3 days to remove the nonvolatile solvents, and the extract was decanted every 8 h and fresh EtOH was replaced. The completely activated sample was obtained by heating the solvent-exchanged sample at 25°C for 2 h and then 120°C for 8 h under a dynamic high vacuum.

Adsorption selectivity

Ideal adsorbed solution theory (IAST) [4, 5] was used to predict binary mixture adsorption from the experimental pure-gas isotherms. To perform the integrations required by IAST, the single-component isotherms should be fitted by a proper model. There is no restriction on the choice of the model to fit the adsorption isotherm, but data over the pressure range under study should be fitted very precisely.[6, 7] The dual-site Langmuir-Freundlich equation were used to fit the experimental data:

$$q = q_{m1} \cdot \frac{b_1 \cdot p^{1/n1}}{1+b_1 \cdot p^{1/n1}} + q_{m2} \cdot \frac{b_2 \cdot p^{1/n2}}{1+b_2 \cdot p^{1/n2}} \qquad (1)$$

Here, P is the pressure of the bulk gas at equilibrium with the adsorbed phase (kPa), q is the adsorbed amount per mass of adsorbent (mol/kg), q_{m1} and q_{m2} are the saturation capacities of sites 1 and 2 (mol/kg), b_1 and b_2 are the affinity coefficients of sites **1** (1/kPa), and n_1 and n_2 represent the deviations from an ideal homogeneous surface. The R_2 values for all the fitted isotherms were over 0.99999. Hence, the fitted isotherm parameters were applied to perform the necessary integrations in IAST.

Estimation of the isosteric heats of gas adsorption

A virial-type expression comprising the temperature-independent parameters a_i and b_i was employed to calculate the enthalpies of adsorption for C_2H_2, C_2H_4 and C_2H_6 (at 273, 298 and 308 K) on **NTU-96** to **NTU-98**. In each case, the data were fitted using the equation (2):

$$Ln\, P = Ln\, N + 1/T \sum_{i=0}^{m} a_i N^i + \sum_{i=0}^{n} b_i N^i \qquad (2)$$

Here, P is the pressure expressed in Torr, N is the amount adsorbed in mmol g^{-1}, T is the temperature in K, a_i and b_i are virial coefficients, and m, n represent the number of coefficients required to adequately describe the isotherms (m and n were gradually increased until the contribution of extra added a and b coefficients were deemed to be statistically

insignificant towards the overall fit, and the average value of the squared deviations from the experimental values was minimized).

$$Q_{st} = -R \sum_{i=0}^{m} a_i N^i \qquad (3)$$

Here, Q_{st} is the coverage-dependent isosteric heat of adsorption and R is the universal gas constant (3).

Dispersion-corrected density functional theory calculations

Density functional theory (DFT) calculations were conducted using the Quantum-Espresso package.[8] A semiempirical addition of dispersive forces to conventional DFT, “Grimme-D3”, was included in the calculation to account for van der Waals interactions.[9] We used Vanderbilt-type ultrasoft pseudopotentials and generalized gradient approximation (GGA) with a Perdew−Burke−Ernzerhof (PBE) exchange correlation. A cutoff energy of 680 eV and a 2 × 2 × 2 k-point mesh (generated using the Monkhosrt-Pack scheme) were found to be enough for the total energy to converge within 0.01 meV/atom. First, the bare MOF structure was fully optimized, using the primitive cell. For gas adsorption, the guest molecule was introduced into the MOF structure, with various possible binding configurations (in terms of both the location and orientation of the guest gas molecule) considered, and then fully relaxed. The lowest-energy structures were identified as the optimal binding structures at various adsorption sites. To obtain the gas binding energies, a single gas molecule placed in a supercell with the same cell dimensions was also relaxed as a reference. The static binding energy (at T = 0 K) was calculated using: $E_B = E(\text{MOF}) + E(\text{gas}) - E(\text{MOF+gas})$.

Supplementary Table 1. Crystal data and structure refinement.

**Compound**	**Empirical formula**	**Formula weight**	**Space group**	***a* / Å**	***b* / Å**	***c* / Å**	***β* / °**	**V / Å³**	***Z***
**NTU-96**	$C_{33}H_{12}F_{24}N_{12}O_{13}Zn_4$	1502.05	*Fm-3m*	20.2115(5)	20.2115(5)	20.2115(5)	90	8256.5(6)	8
**NTU-97**	$C_{15}H_{12}N_6O_7Zn_4$	649.79	*Fm-3m*	20.1655(10)	20.1655(10)	20.1655(10)	90	8200.3(12)	8
**NTU-98**	$C_{18}H_{18}N_6O_7Zn_4$	691.94	*F-43m*	20.1691(5)	20.1691(5)	20.1691(5)	90	8204.6(6)	8
**NTU-98⊃C_2H_2**	$C_{152}H_{152}N_{48}O_{56}Zn_{32}$	5634.88	*Fm-3m*	20.1249(3)	20.1249(3)	20.1249(3)	90	8150.8(4)	1
**NTU-98⊃C_2H_4**	$C_{152}H_{160}N_{48}O_{56}Zn_{32}$	5642.62	*Fm-3m*	20.1114(4)	20.1114(4)	20.1114(4)	90	8134.4(5)	1
**NTU-98⊃C_2H_6**	$C_{152}H_{168}N_{48}O_{56}Zn_{32}$	5659.54	*Fm-3m*	20.1454(4)	20.1454(4)	20.1454(4)	90	8175.8(5)	1

$R_1 = \Sigma||F_o|-|F_c||/\Sigma|F_o|$, $wR_2 = [\Sigma w(\Sigma Fo^2 - Fc^2)^2/\Sigma w(Fo^2)^2]^{1/2}$

Supplementary Table 2. Comparison of molecular sizes and physical properties of C_2H_2, C_2H_4 and C_2H_6.[10, 11]

	C_2H_2	C_2H_4	C_2H_6
Molecular size (Å^3)	3.32 × 3.34 × 5.70	3.28 × 4.18 × 4.84	3.81 × 4.08 × 4.82
Boiling point (K)	189.3	169.5	184.6
Polarizability (×10^{-25} cm^3)	33.3-39.3	42.5	44.3-44.7
Kinetic diameter (Å)	3.3	4.2	4.4

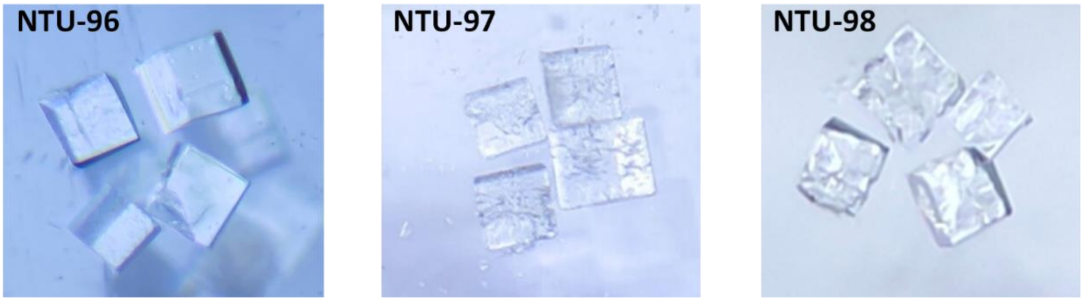

Supplementary Fig. 1. Optical photos of **NTU-96**, **NTU-97** and **NTU-98**.

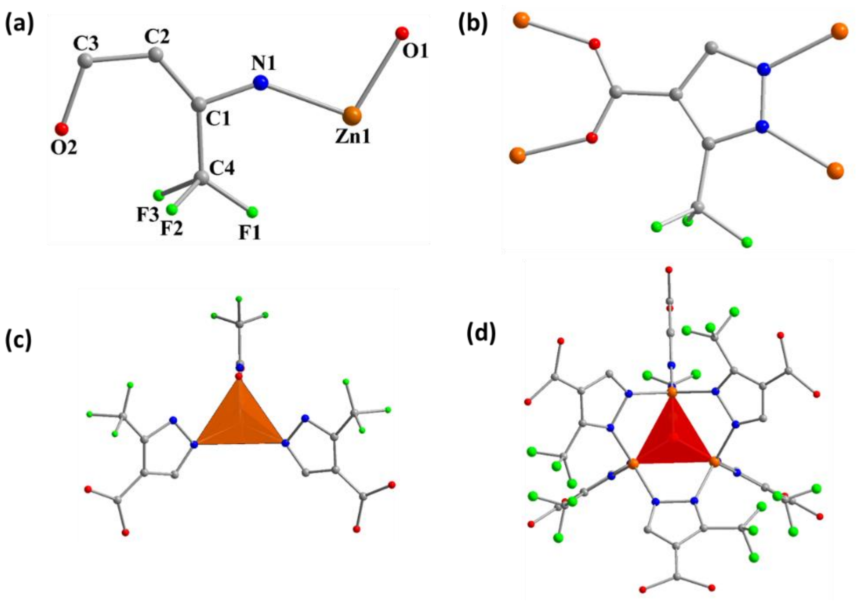

Supplementary Fig. 2. Structure of **NTU-96**: (a) Asymmetric unit; (b) Ligand connection; (c) Zn central connection; (d) Zn_4O configuration. Color code: C, gray; N, blue; O, red; F, green; The orange polyhedron represents zinc atoms with tetrahedral coordination..

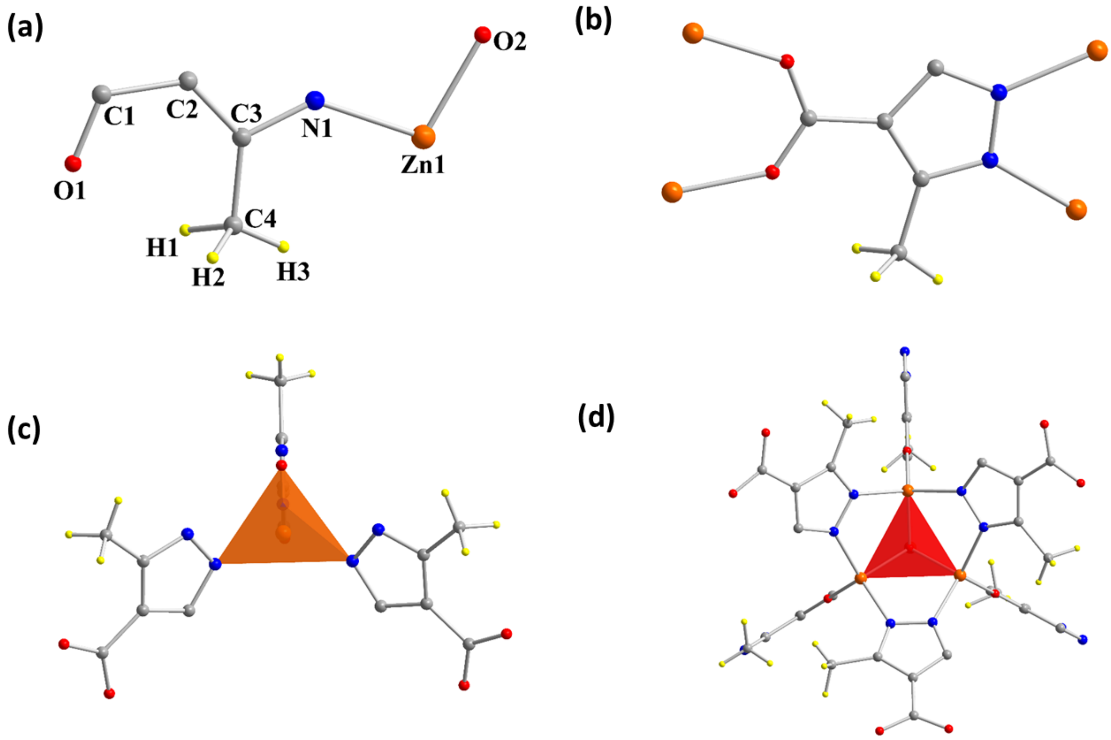

Supplementary Fig. 3. Structure of **NTU-97**: (a) Asymmetric unit; (b) Ligand connection; (c) Zn central connection; (d) Zn_4O configuration. Color code: C, gray; N, blue; O, red; F, green; The orange polyhedron represents zinc atoms with tetrahedral coordination..

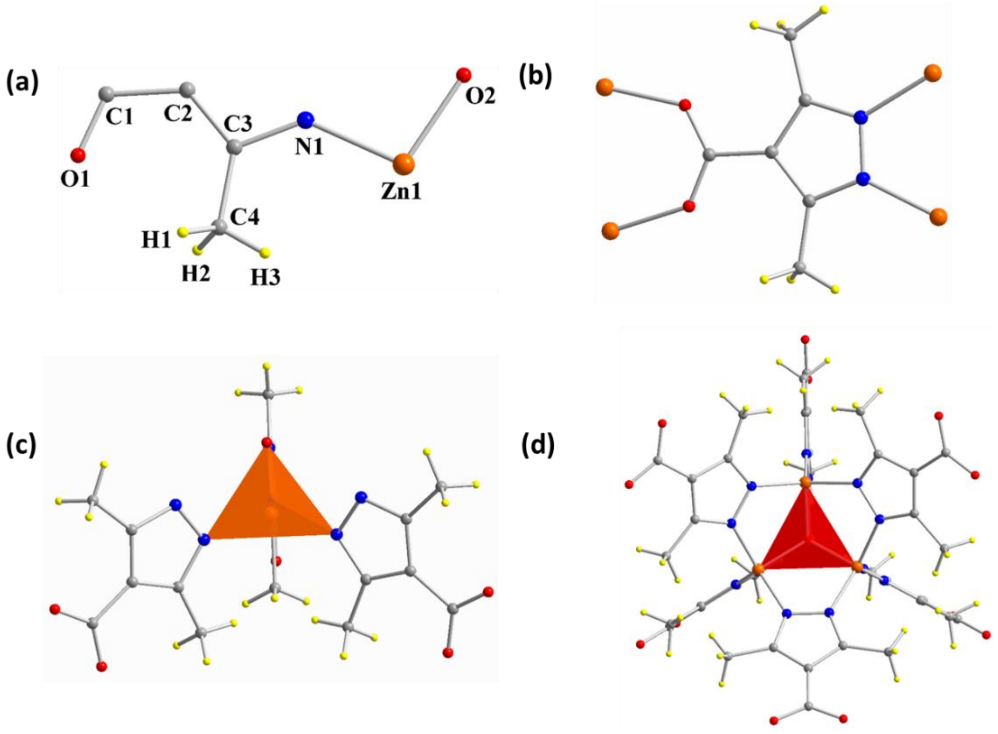

Supplementary Fig. 4. Structure of **NTU-98**: (a) Asymmetric unit; (b) Ligand connection; (c) Zn central connection; (d) Zn_4O configuration. Color code: C, gray; N, blue; O, red; F, green; The orange polyhedron represents zinc atoms with tetrahedral coordination..

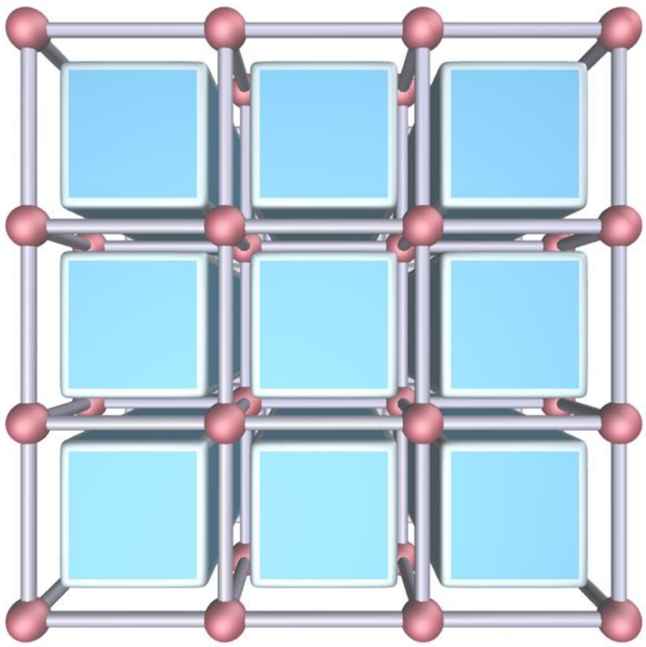

Supplementary Fig. 5. View of the **pcu** topology of **NTU-96 to NTU-98**.

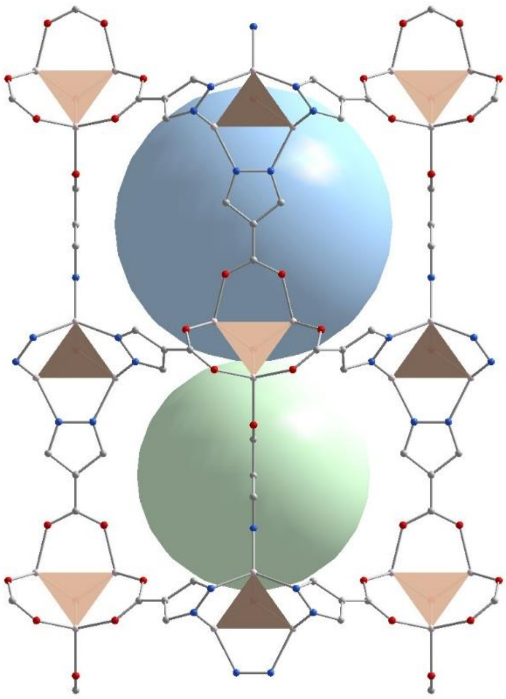

Supplementary Fig. 6. View of the two cages of $Zn_4O(PyC)_3$. Although it is a MOF-5 analog, the coordination of the two N sites of 4-PyC locks the direction of the ligand, yielding the three-dimensional extension with two different cages (8.0 and 10.8 Å).

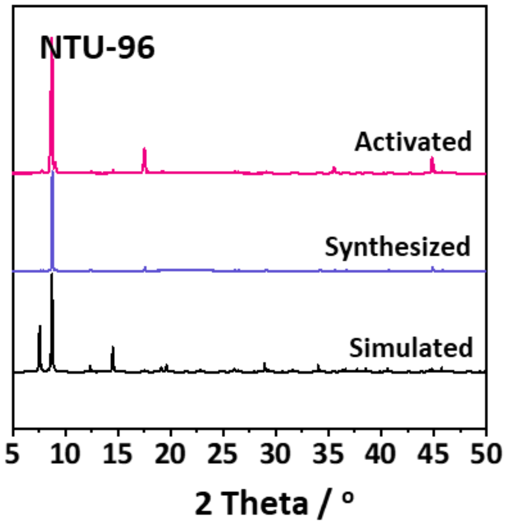

Supplementary Fig. 7. PXRD patterns of **NTU-96**.

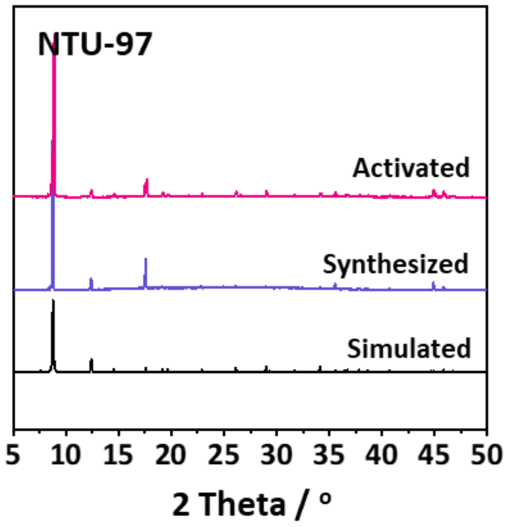

Supplementary Fig. 8. PXRD patterns of **NTU-97**.

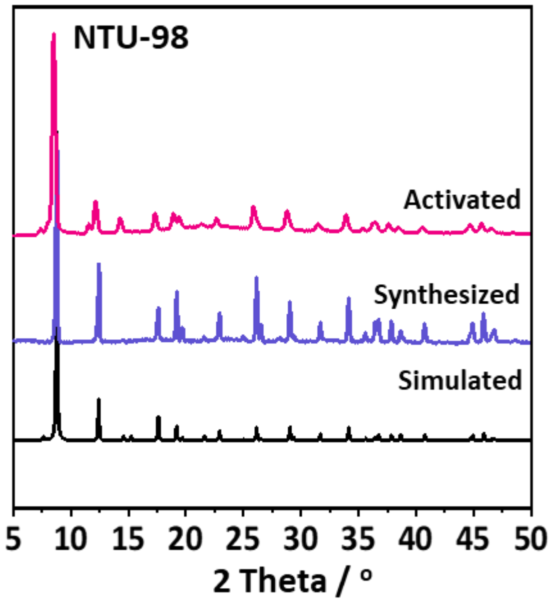

Supplementary Fig. 9. PXRD patterns of **NTU-98**.

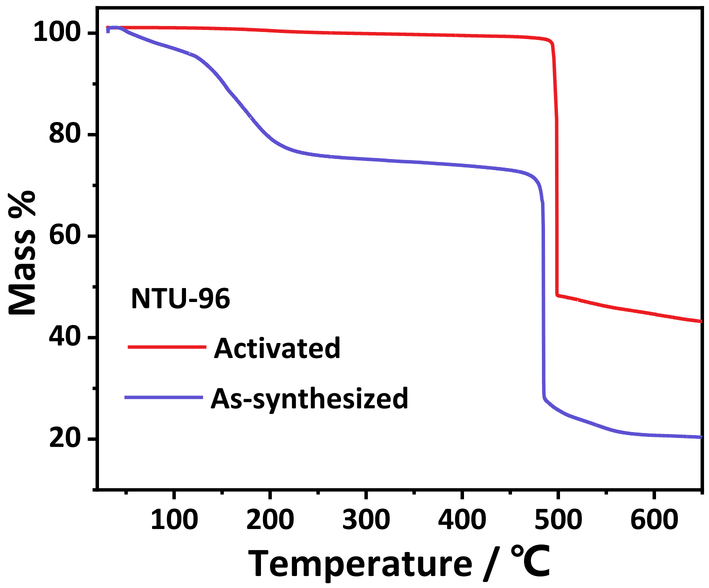

Supplementary Fig. 10. TG curves of **NTU-96**.

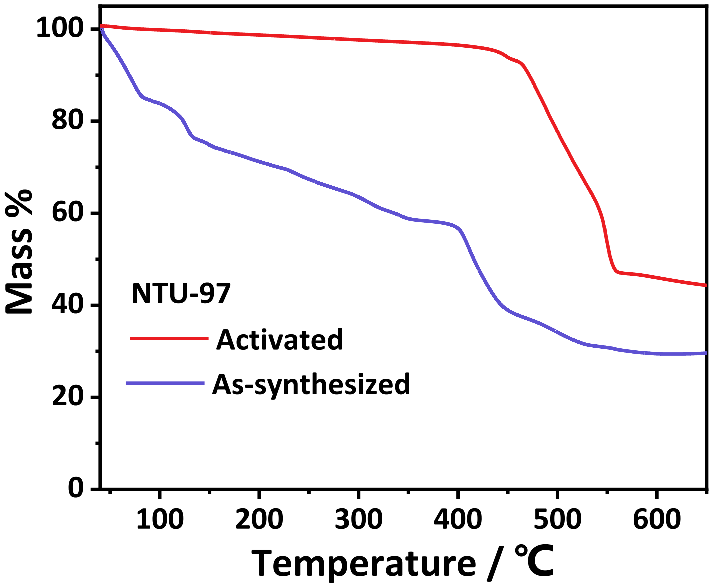

Supplementary Fig. 11. TG curves of **NTU-97**.

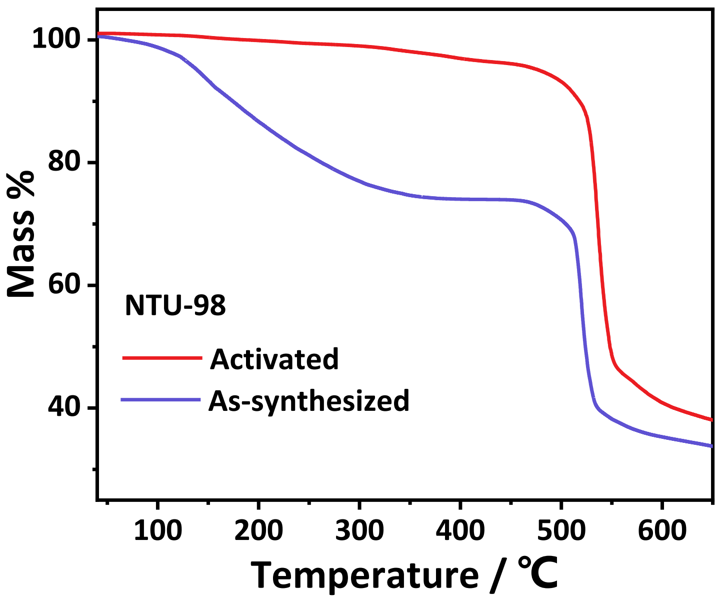

Supplementary Fig. 12. TG curves of **NTU-98**.

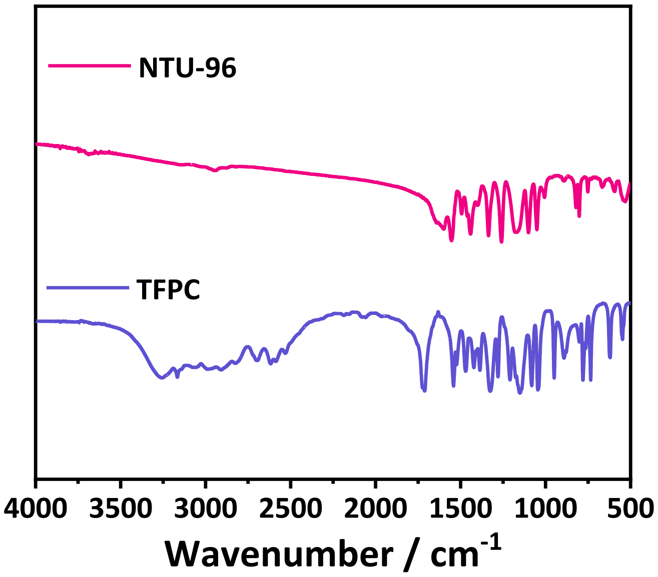

Supplementary Fig. 13. FTIR of **TFPC** ligand and **NTU-96**.

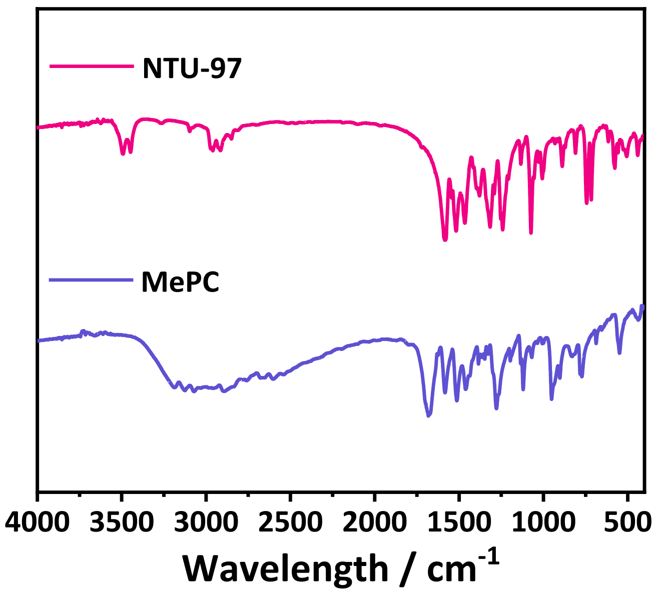

Supplementary Fig. 14. FTIR of **MePC** ligand and **NTU-97**.

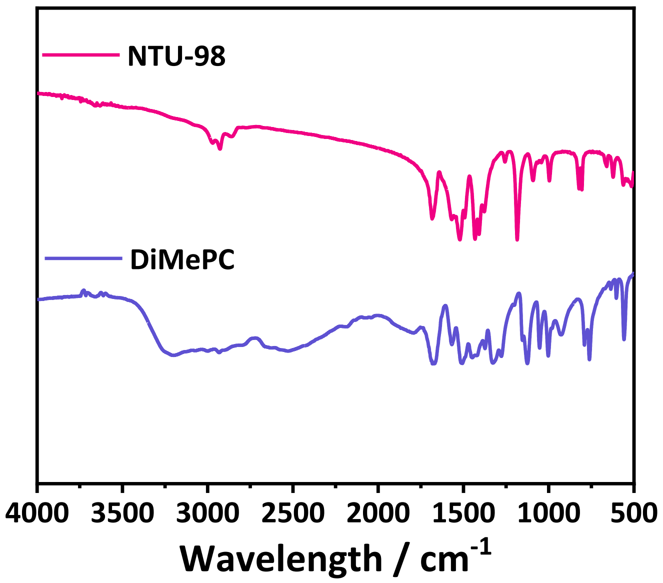

Supplementary Fig. 15. FTIR of **DiMePC** ligand and **NTU-98**.

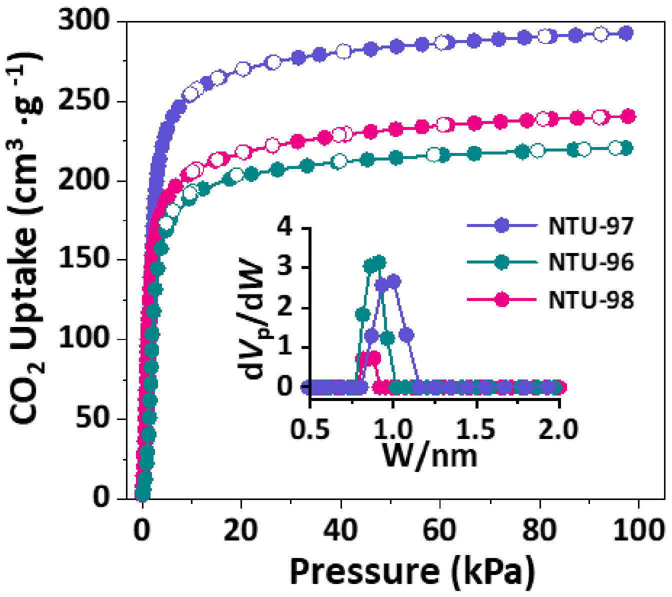

Supplementary Fig. 16. CO_2 adsorption isotherms at 195 K and pore size distribution of **NTU-96**, **NTU-97** and **NTU-98**.

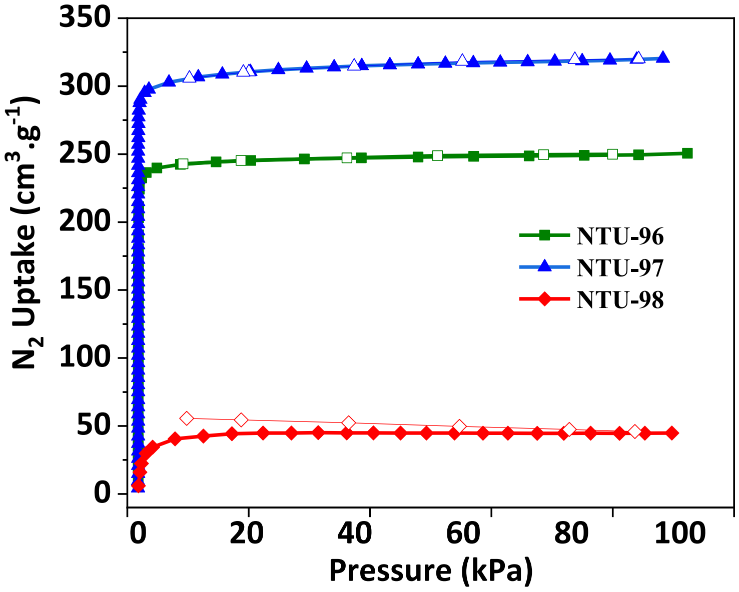

Supplementary Fig. 17. N_2 adsorption isotherms at 77 K and pore size distribution of **NTU-96**, **NTU-97** and **NTU-98**.

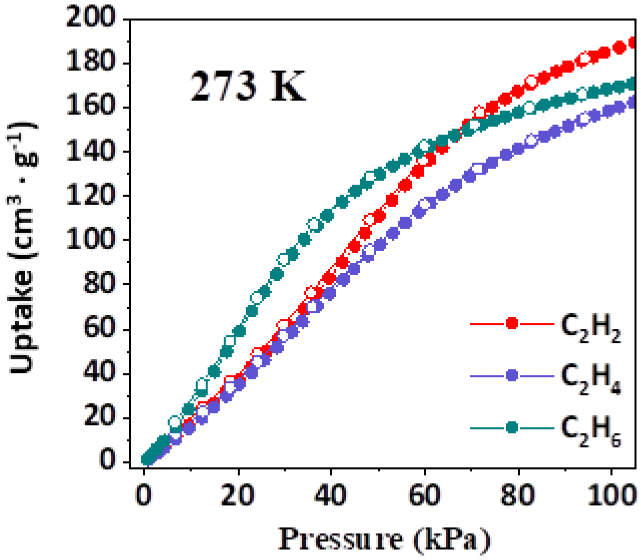

Supplementary Fig. 18. Adsorption isotherms of C_2H_2, C_2H_4 and C_2H_6 for $Zn_4O(PyC)_3$ at 273 K.

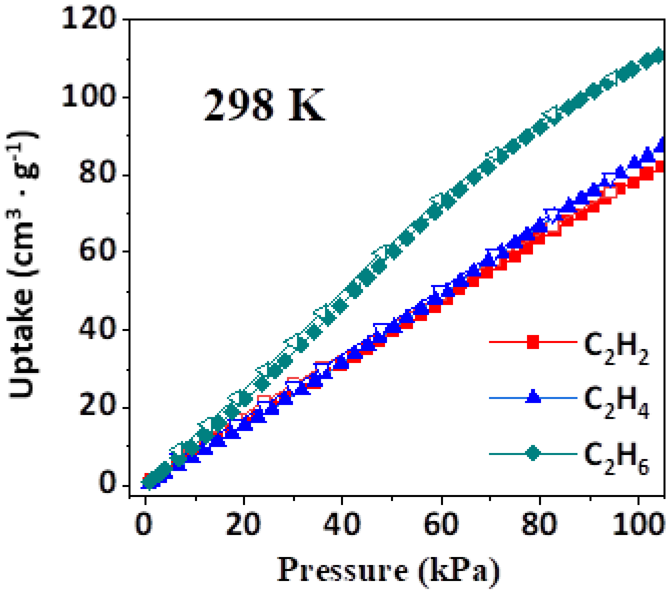

Supplementary Fig. 19. Adsorption isotherms of C_2H_2, C_2H_4 and C_2H_6 for $Zn_4O(PyC)_3$ at 298 K.

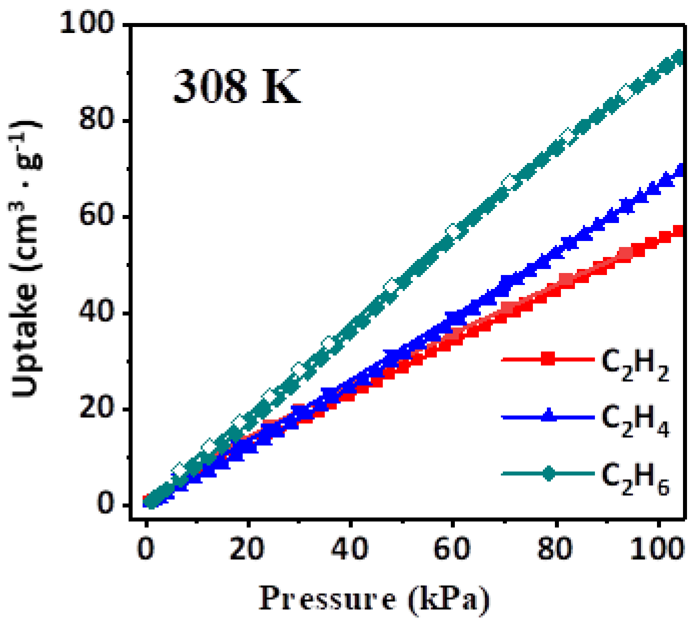

Supplementary Fig. 20. Adsorption isotherms of C_2H_2, C_2H_4 and C_2H_6 for $Zn_4O(PyC)_3$ at 308 K.

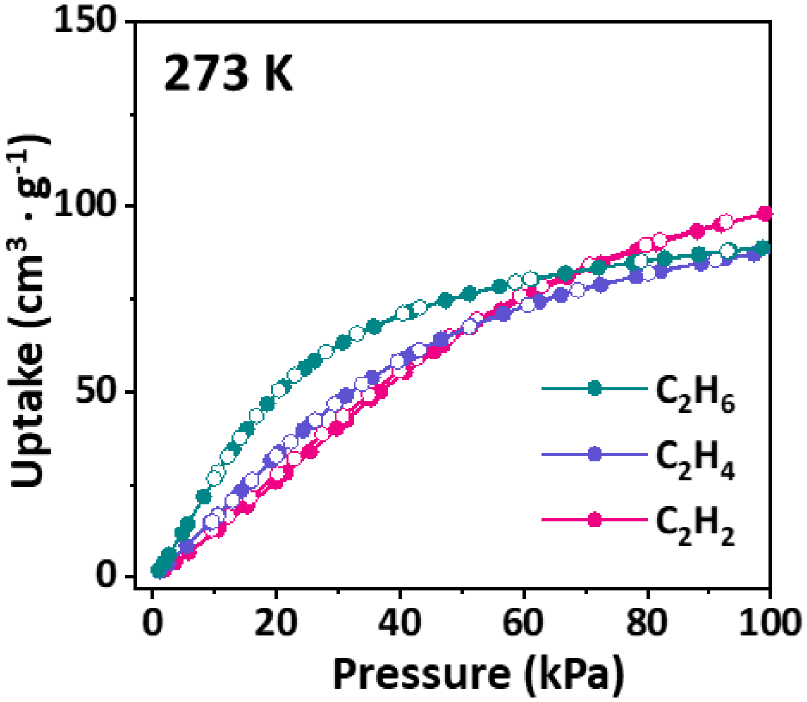

Supplementary Fig. 21. Adsorption isotherms of C_2H_2, C_2H_4 and C_2H_6 on **NTU-96** at 273 K.

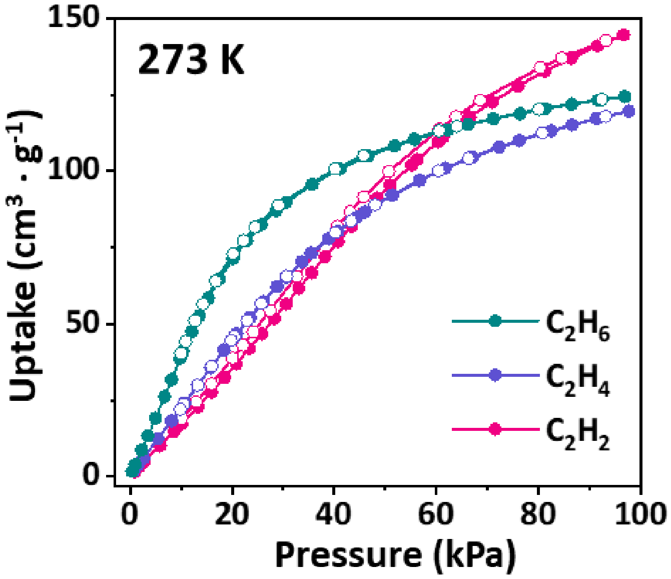

Supplementary Fig. 22. Adsorption isotherms of C_2H_2, C_2H_4 and C_2H_6 on **NTU-97** at 273 K.

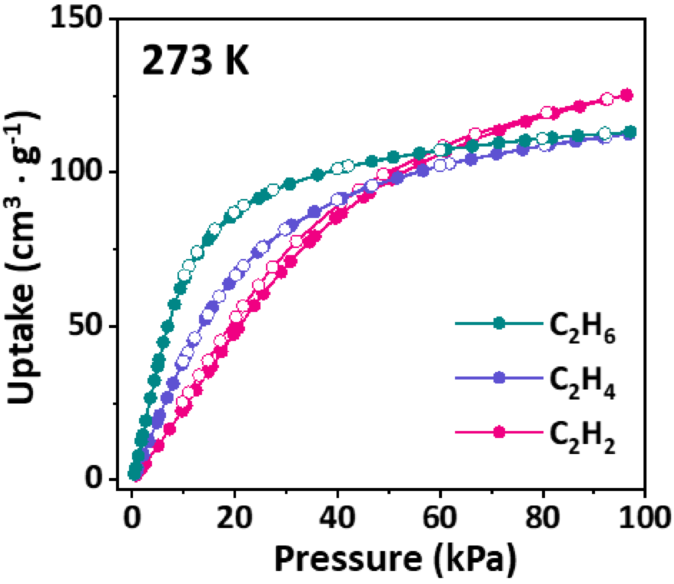

Supplementary Fig. 23. Adsorption isotherms of C_2H_2, C_2H_4 and C_2H_6 on **NTU-98** at 273 K.

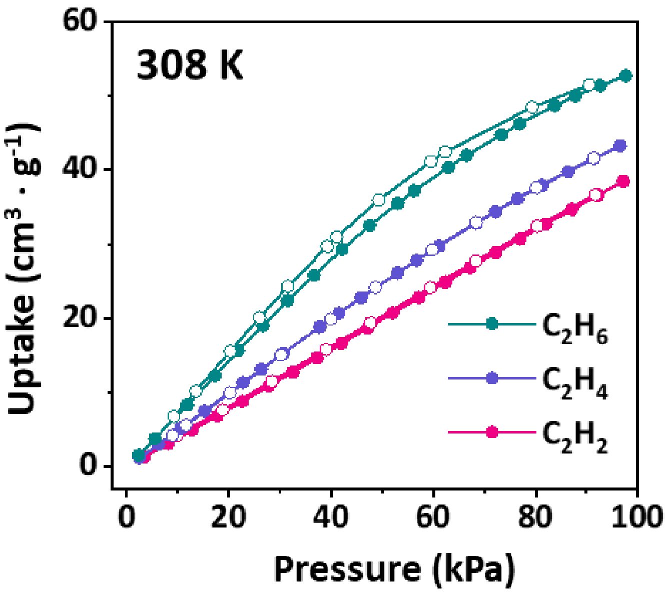

Supplementary Fig. 24. Adsorption isotherms of C_2H_2, C_2H_4 and C_2H_6 on **NTU-96** at 308 K.

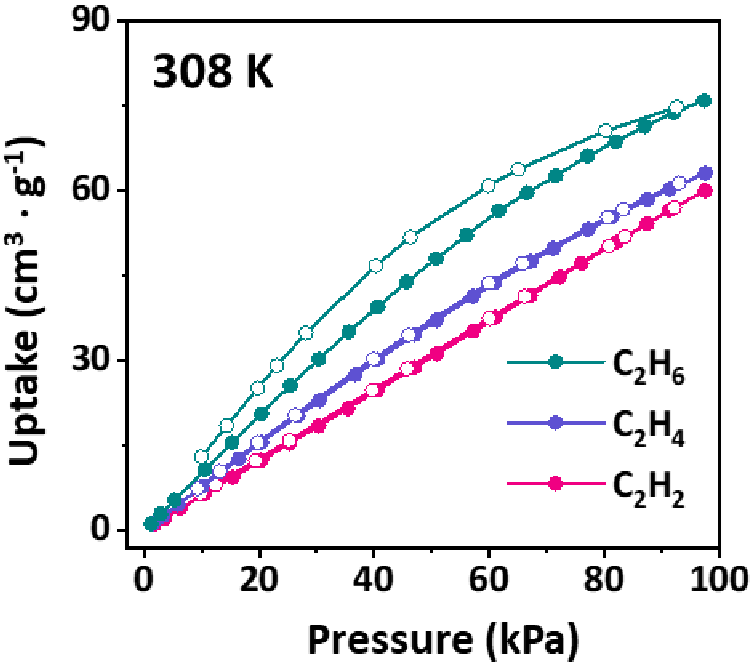

Supplementary Fig. 25. Adsorption isotherms of C_2H_2, C_2H_4 and C_2H_6 on **NTU-97** at 308 K.

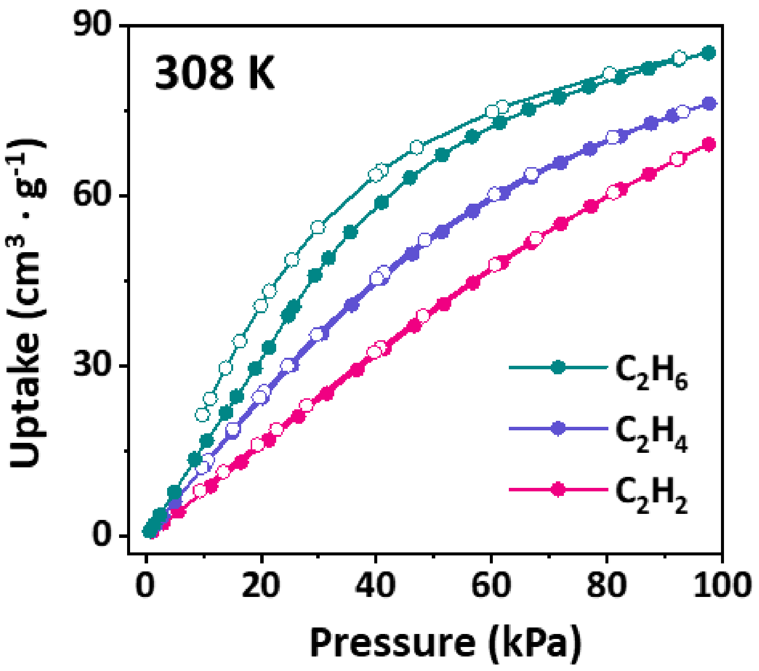

Supplementary Fig. 26. Adsorption isotherms of C_2H_2, C_2H_4 and C_2H_6 on **NTU-98** at 308 K.

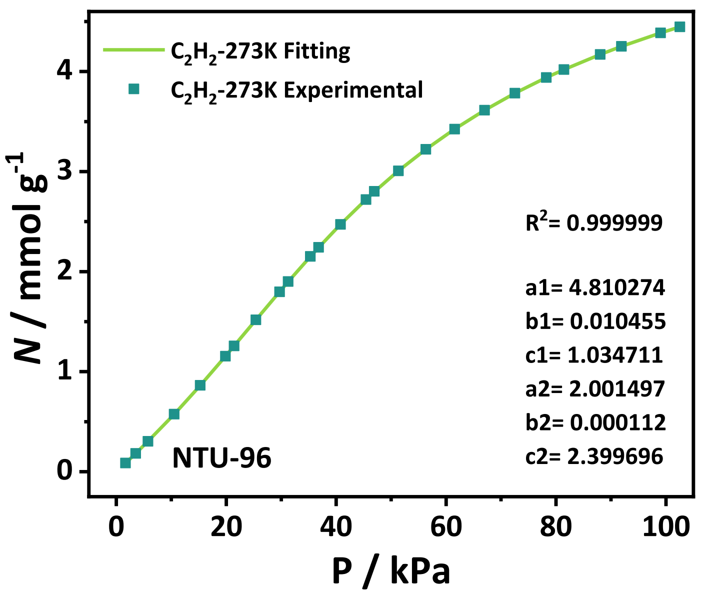

Supplementary Fig. 27. Fitting of adsorption isotherms for C_2H_2 in **NTU-96** at 273 K.

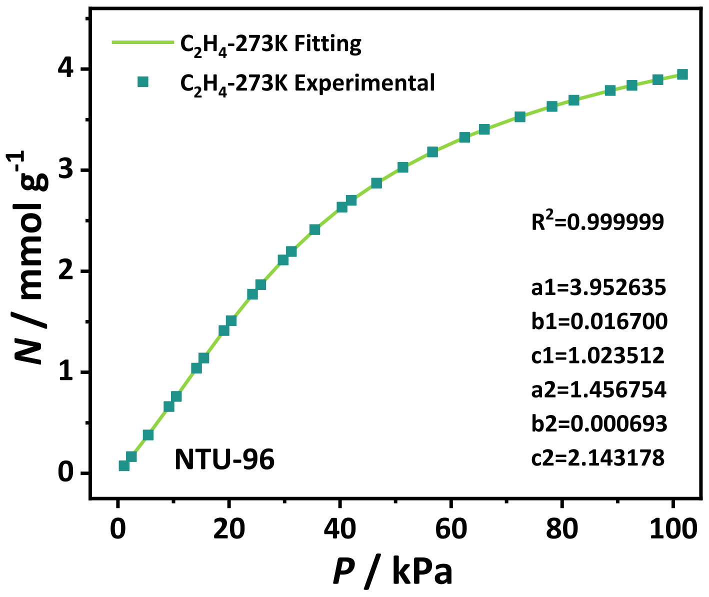

Supplementary Fig. 28. Fitting of adsorption isotherms for C_2H_4 in **NTU-96** at 273 K.

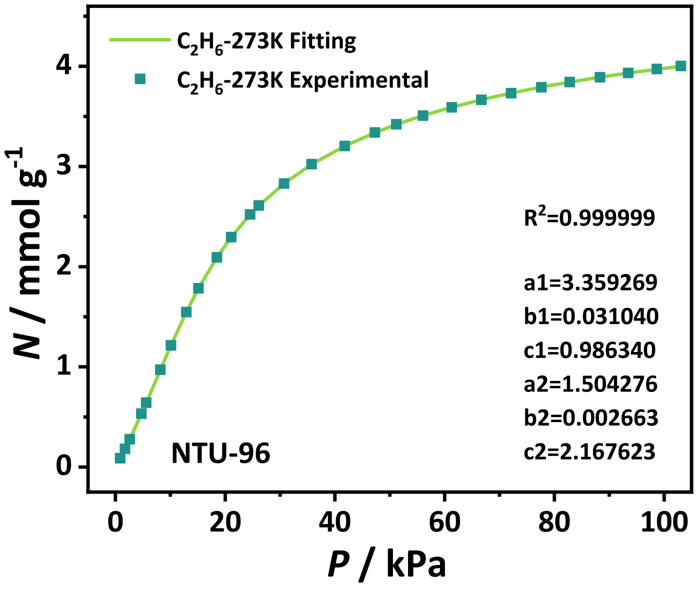

Supplementary Fig. 29. Fitting of adsorption isotherms for C_2H_6 in **NTU-96** at 273 K.

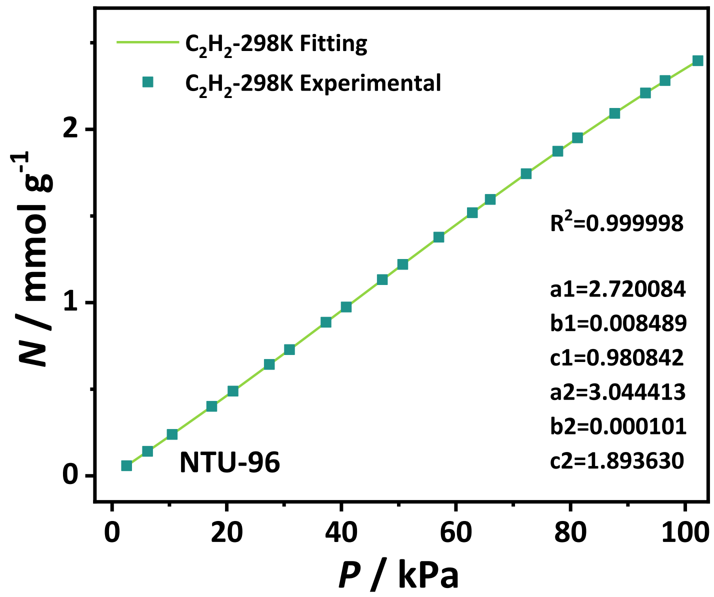

Supplementary Fig. 30. Fitting of adsorption isotherms for C_2H_2 in **NTU-96** at 298 K.

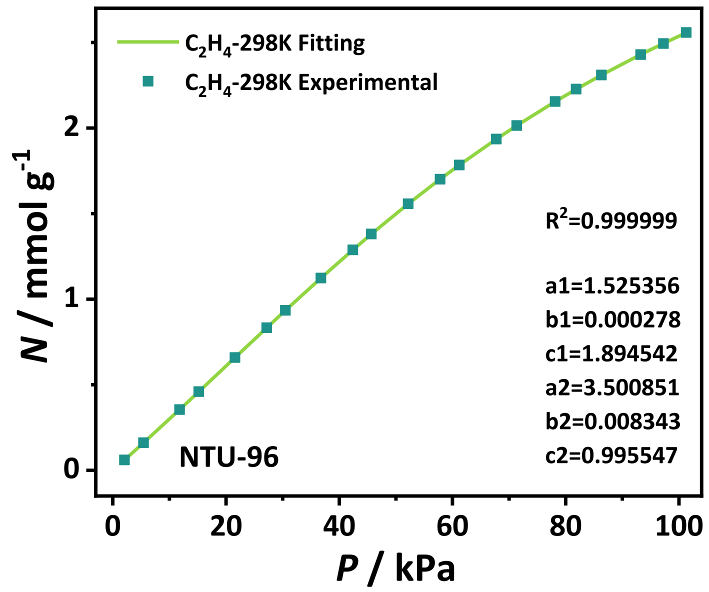

Supplementary Fig. 31. Fitting of adsorption isotherms for C_2H_4 in **NTU-96** at 298 K.

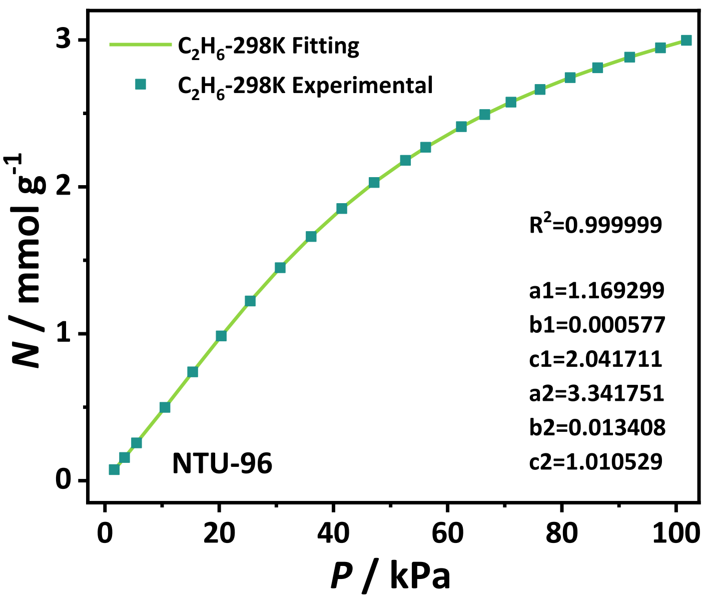

Supplementary Fig. 32. Fitting of adsorption isotherms for C_2H_6 in **NTU-96** at 298 K.

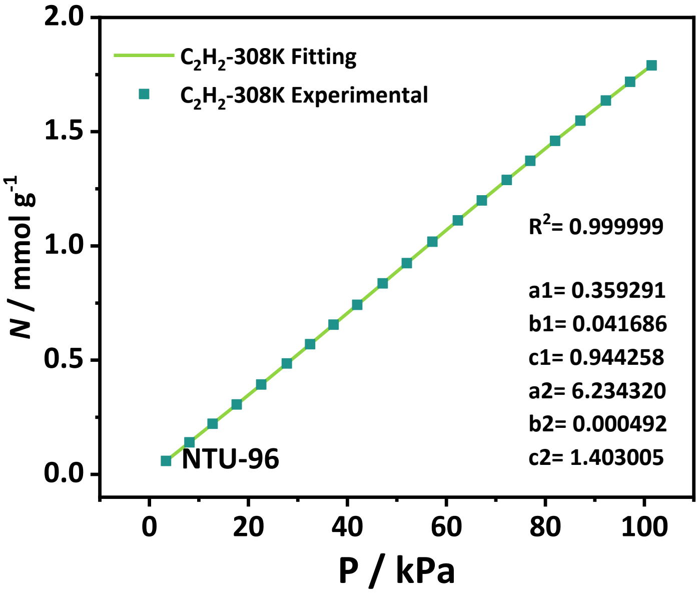

Supplementary Fig. 33. Fitting of adsorption isotherms for C_2H_2 in **NTU-96** at 308 K.

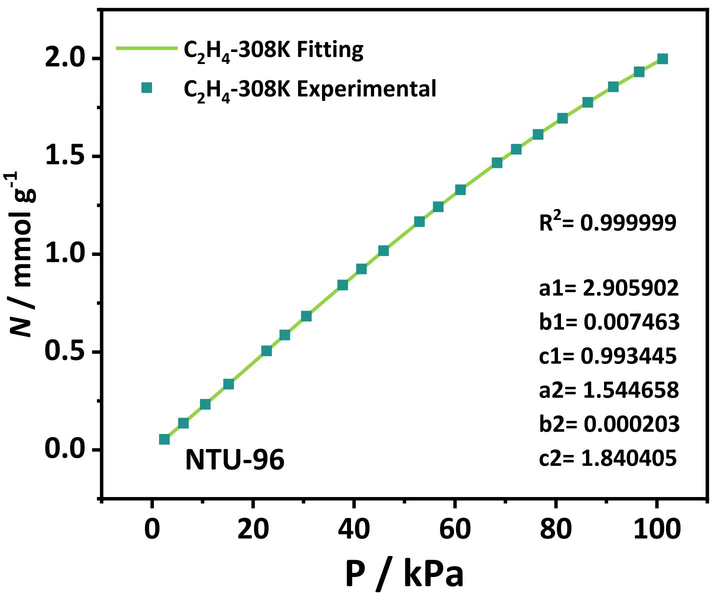

Supplementary Fig. 34. Fitting of adsorption isotherms for C_2H_4 in **NTU-96** at 308 K.

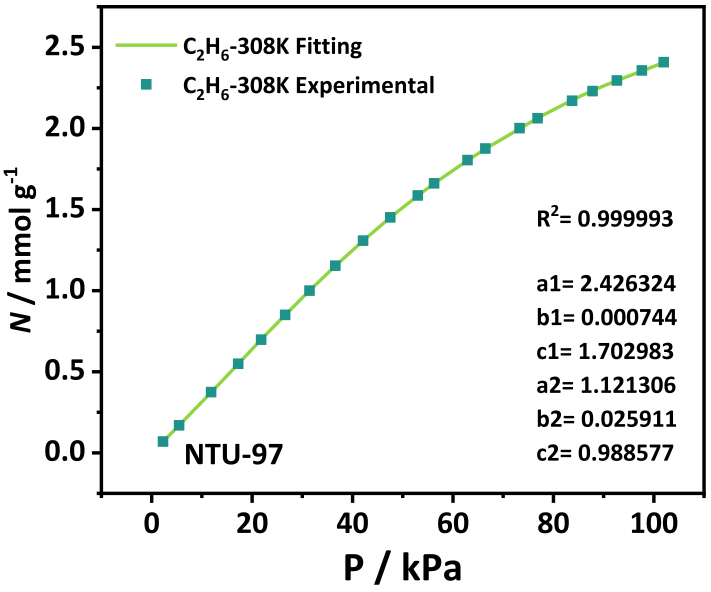

Supplementary Fig. 35. Fitting of adsorption isotherms for C_2H_6 in **NTU-96** at 308 K.

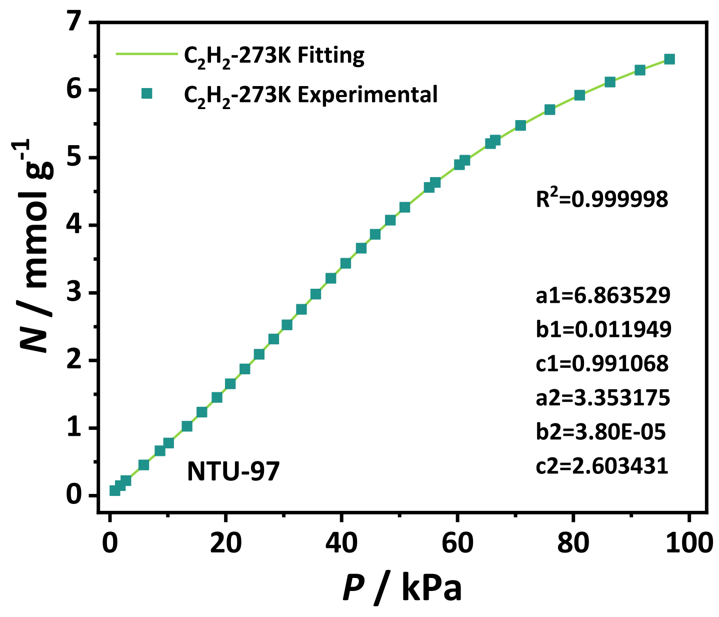

Supplementary Fig. 36. Fitting of adsorption isotherms for C_2H_2 in **NTU-97** at 273 K.

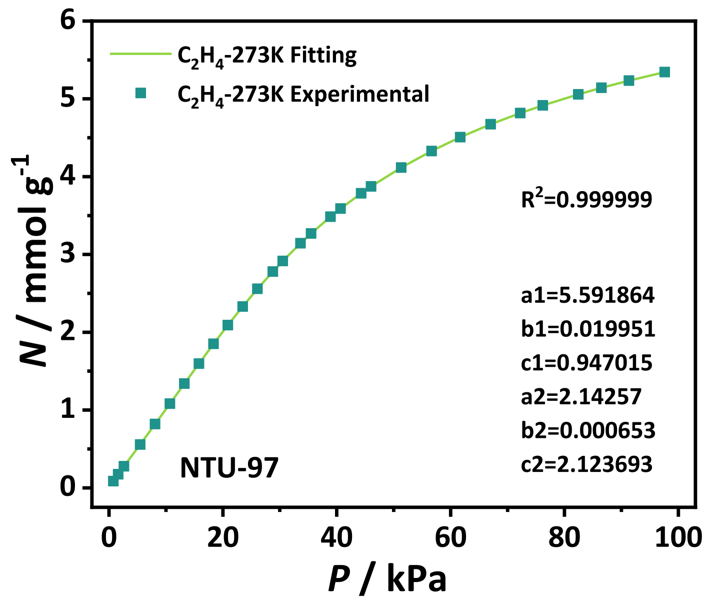

Supplementary Fig. 37. Fitting of adsorption isotherms for C_2H_4 in **NTU-97** at 273 K.

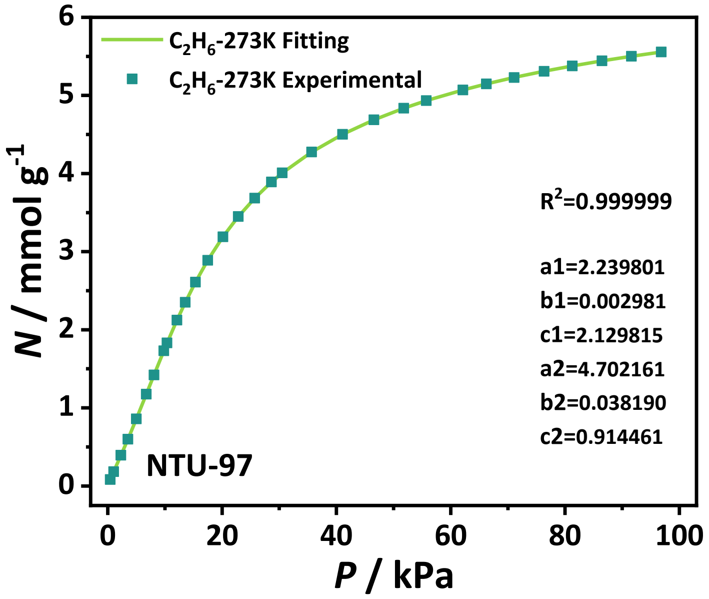

Supplementary Fig. 38. Fitting of adsorption isotherms for C_2H_6 in **NTU-97** at 273 K.

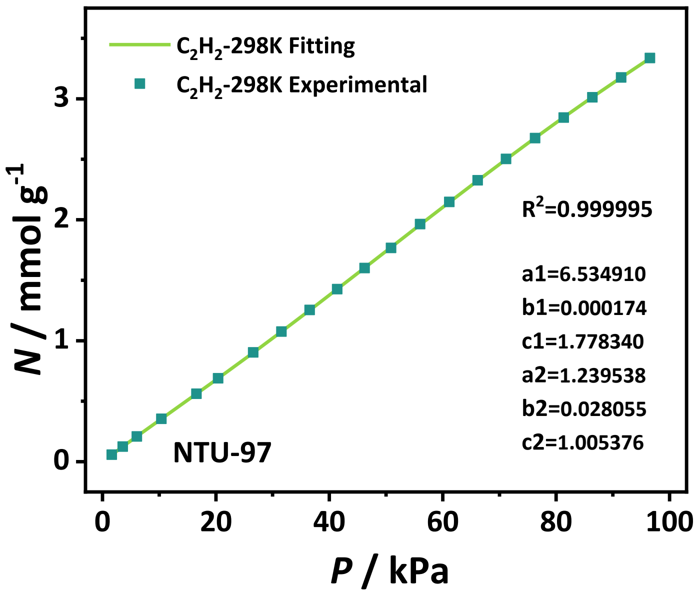

Supplementary Fig. 39. Fitting of adsorption isotherms for C_2H_2 in **NTU-97** at 298 K.

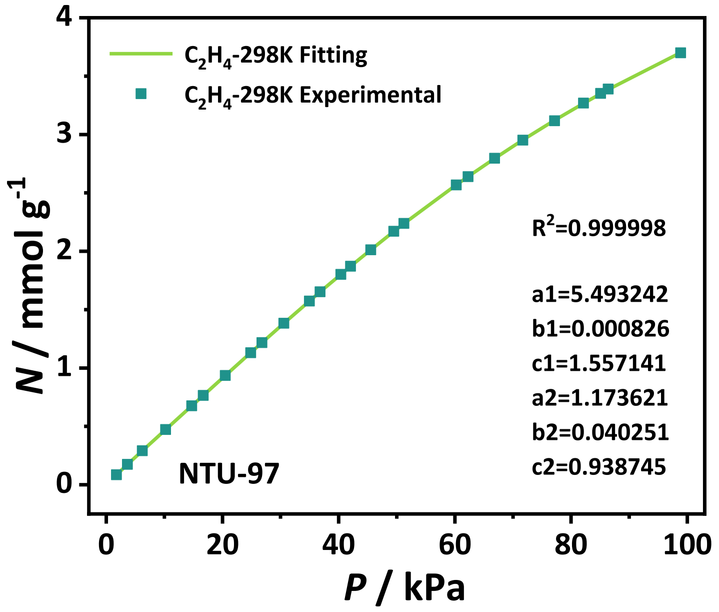

Supplementary Fig. 40. Fitting of adsorption isotherms for C_2H_4 in **NTU-97** at 298 K.

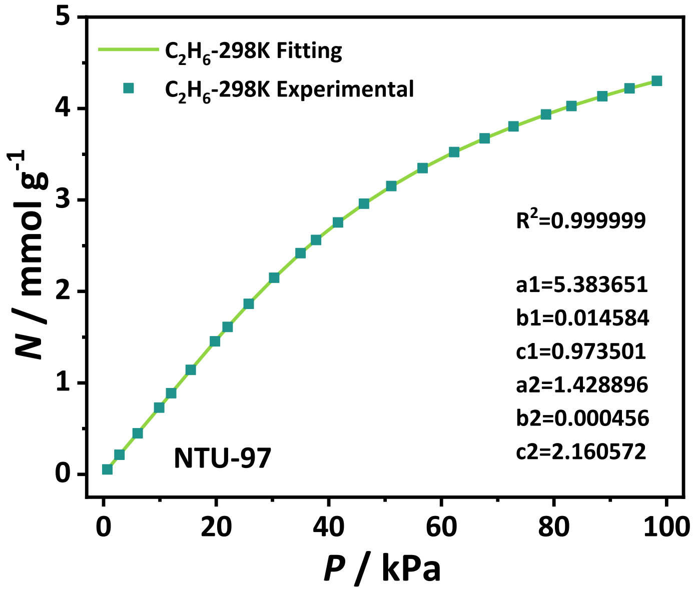

Supplementary Fig. 41. Fitting of adsorption isotherms for C_2H_6 in **NTU-97** at 298 K.

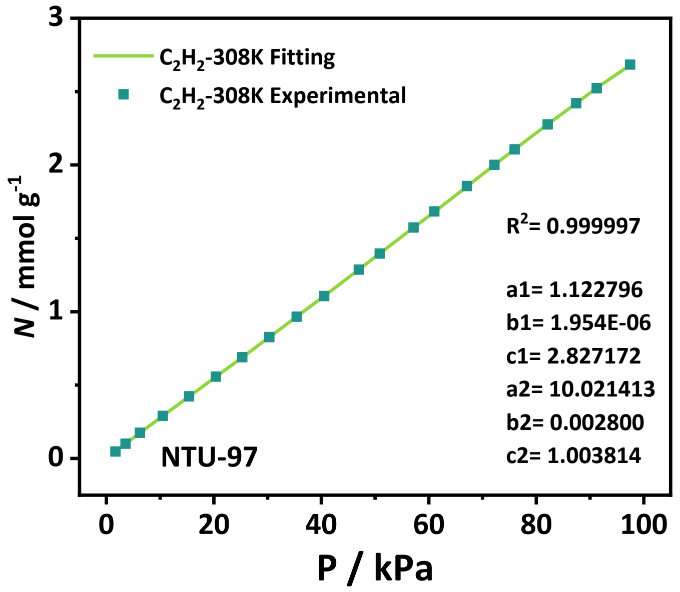

Supplementary Fig. 42. Fitting of adsorption isotherms for C_2H_2 in **NTU-97** at 308 K.

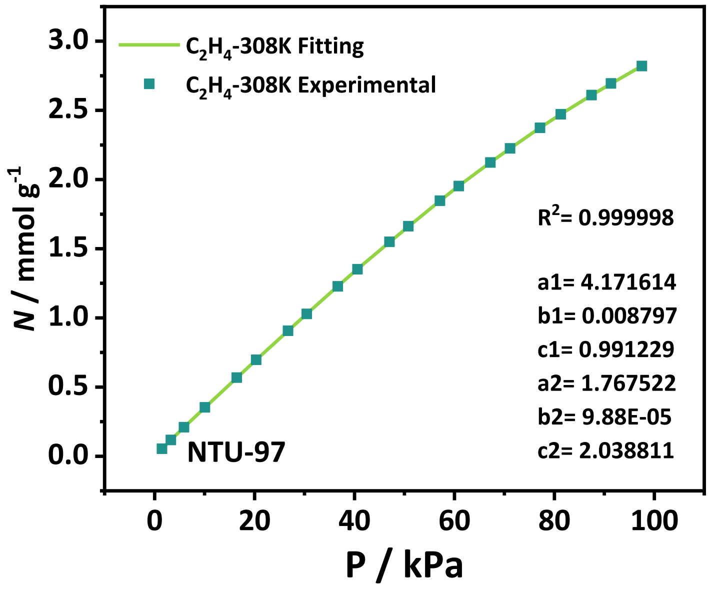

Supplementary Fig. 43. Fitting of adsorption isotherms for C_2H_4 in **NTU-97** at 308 K.

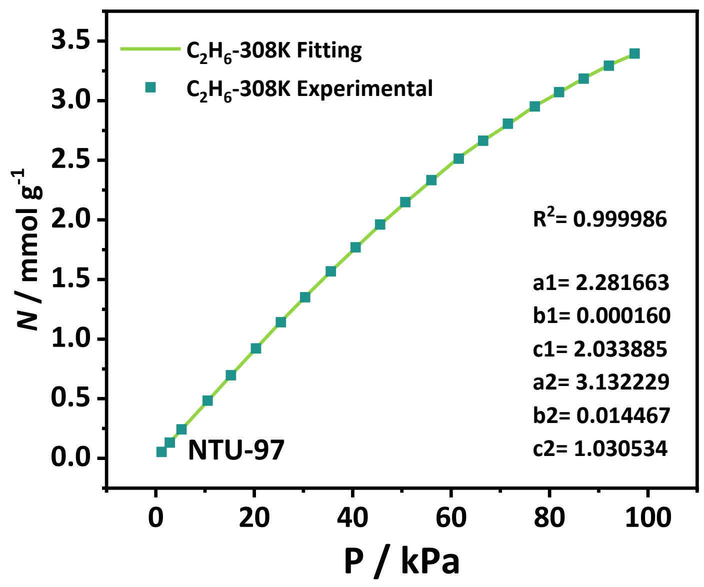

Supplementary Fig. 44. Fitting of adsorption isotherms for C_2H_6 in **NTU-97** at 308 K.

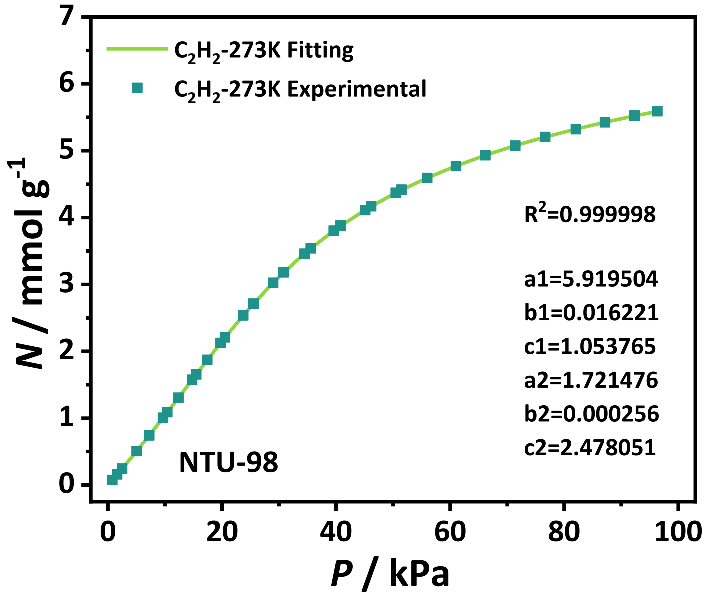

Supplementary Fig. 45. Fitting of adsorption isotherms for C_2H_2 in **NTU-98** at 273 K.

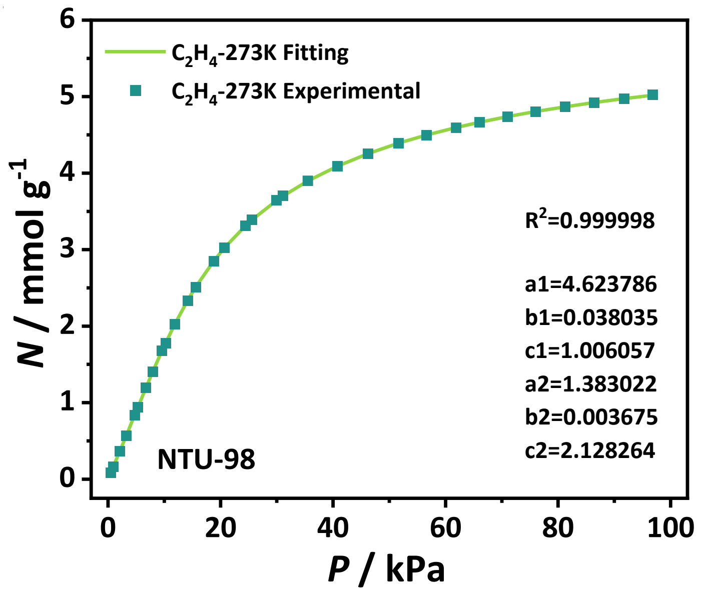

Supplementary Fig. 46. Fitting of adsorption isotherms for C_2H_4 in **NTU-98** at 273 K.

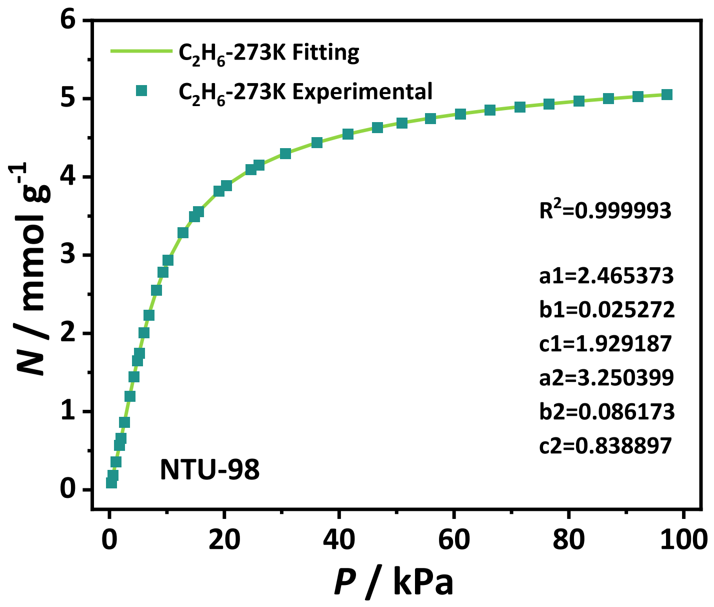

Supplementary Fig. 47. Fitting of adsorption isotherms for C_2H_6 in **NTU-98** at 273 K.

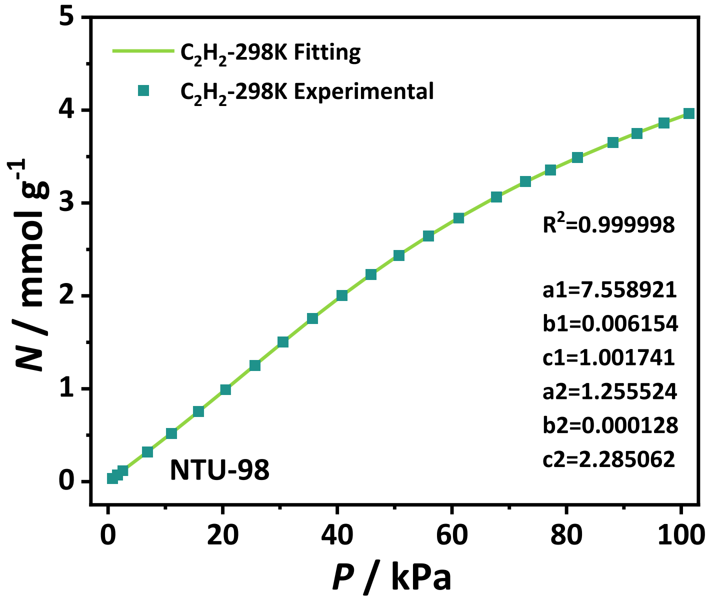

Supplementary Fig. 48. Fitting of adsorption isotherms for C_2H_2 in **NTU-98** at 298 K.

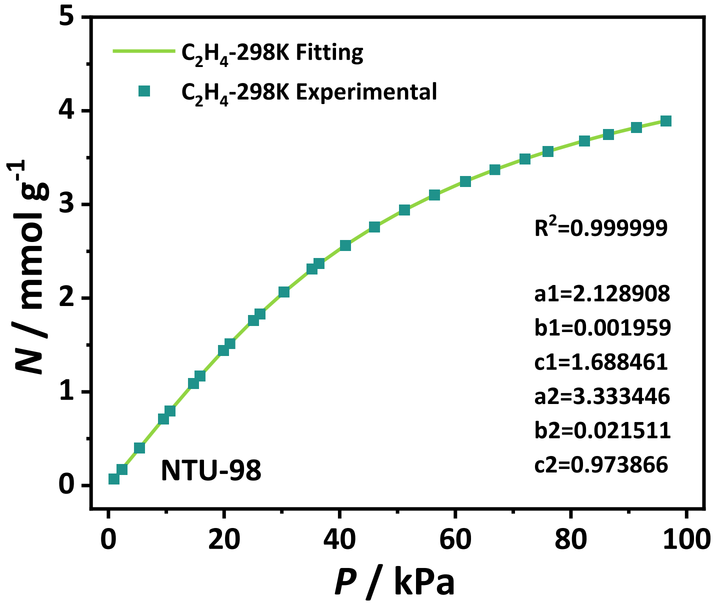

Supplementary Fig. 49. Fitting of adsorption isotherms for C_2H_4 in **NTU-98** at 298 K.

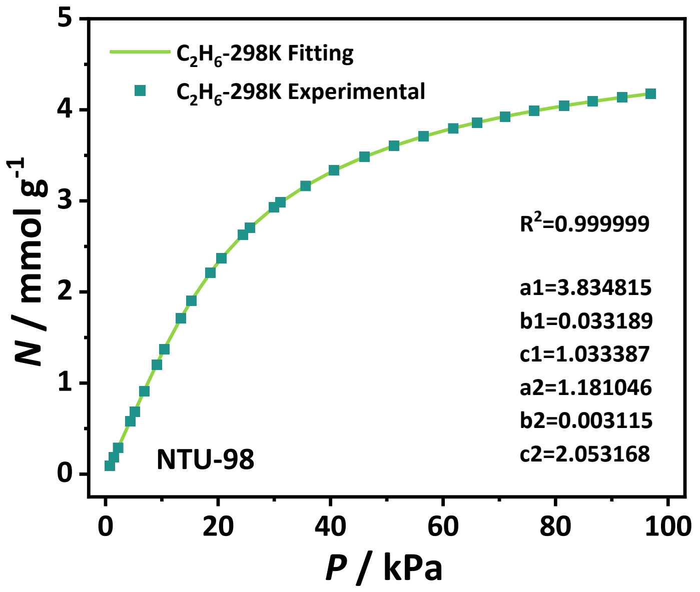

Supplementary Fig. 50. Fitting of adsorption isotherms for C_2H_6 in **NTU-98** at 298 K.

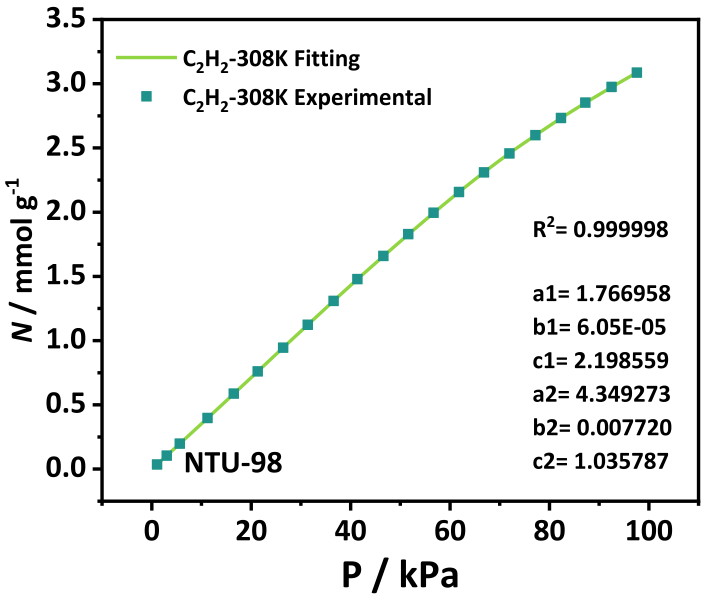

Supplementary Fig. 51. Fitting of adsorption isotherms for C_2H_2 in **NTU-98** at 308 K.

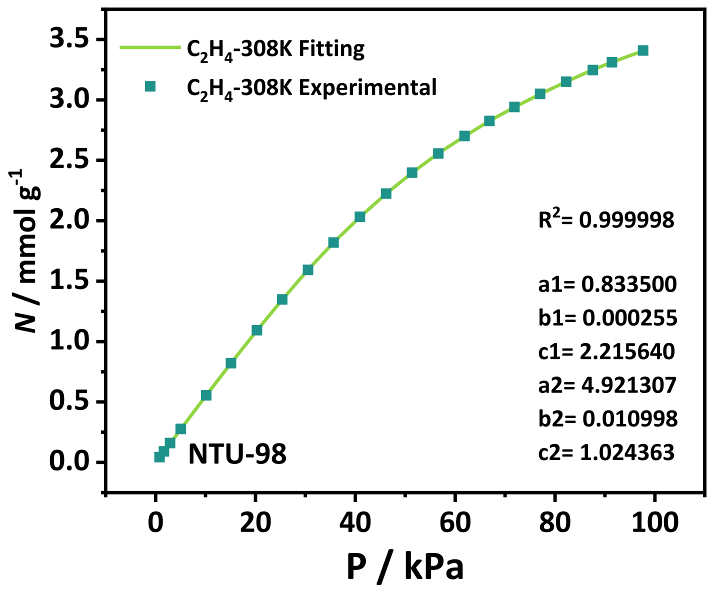

Supplementary Fig. 52. Fitting of adsorption isotherms for C_2H_4 in **NTU-98** at 308 K.

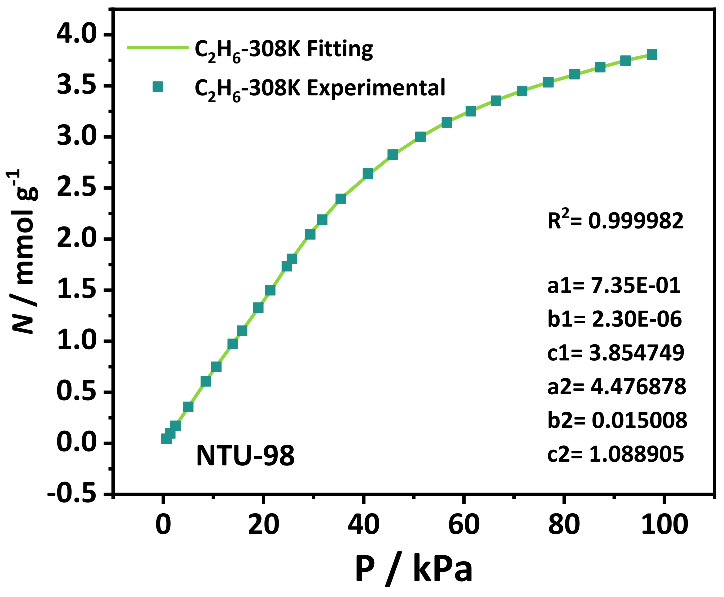

Supplementary Fig. 53. Fitting of adsorption isotherms for C_2H_6 in **NTU-98** at 308 K.

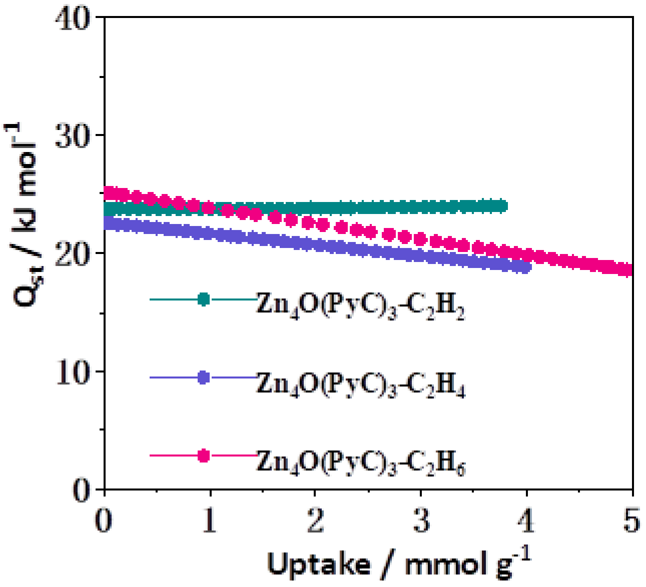

Supplementary Fig. 54. Q_{st} of C_2H_2, C_2H_4 and C_2H_6 for $Zn_4O(PyC)_3$.

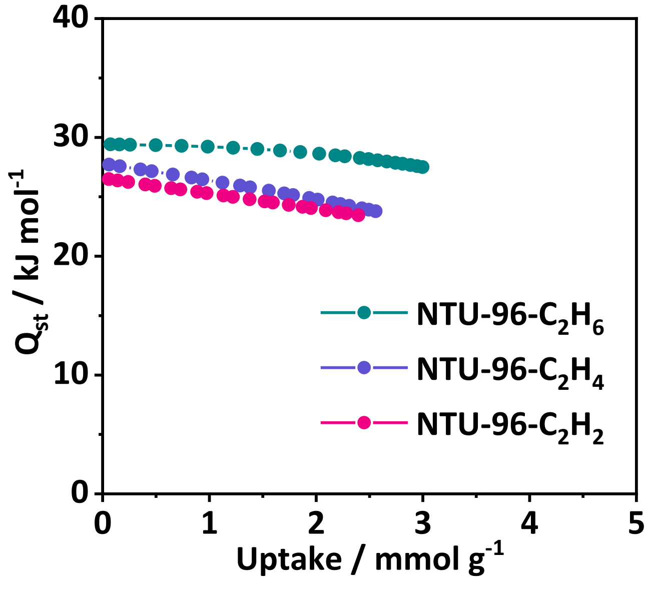

Supplementary Fig. 55. Q_{st} of C_2H_2, C_2H_4 and C_2H_6 for **NTU-96**.

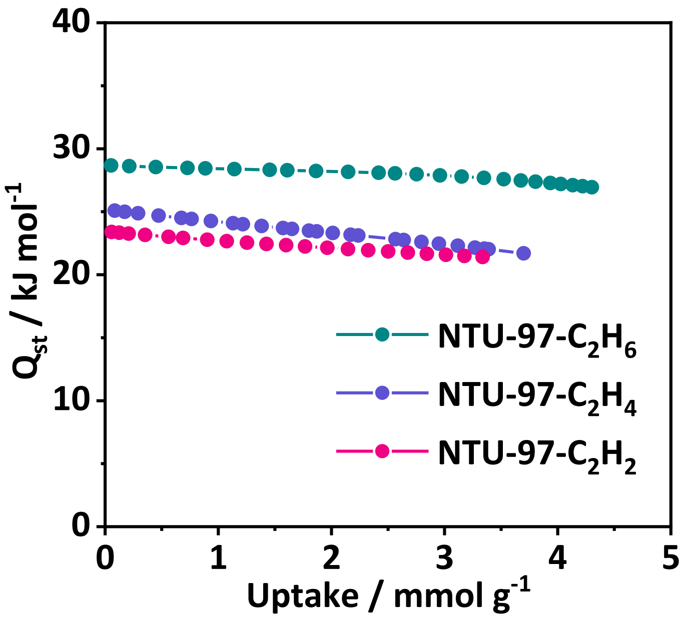

Supplementary Fig. 56. Q_{st} of C_2H_2, C_2H_4 and C_2H_6 for **NTU-97**.

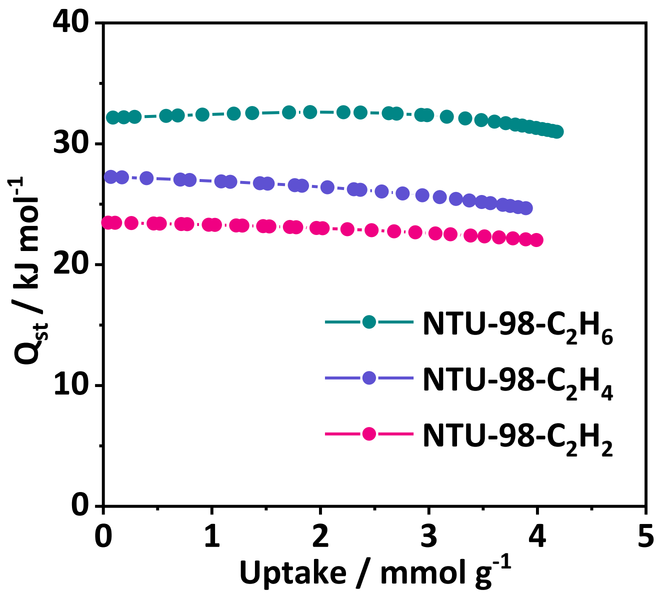

Supplementary Fig. 57. Q_{st} of C_2H_2, C_2H_4 and C_2H_6 for **NTU-98**.

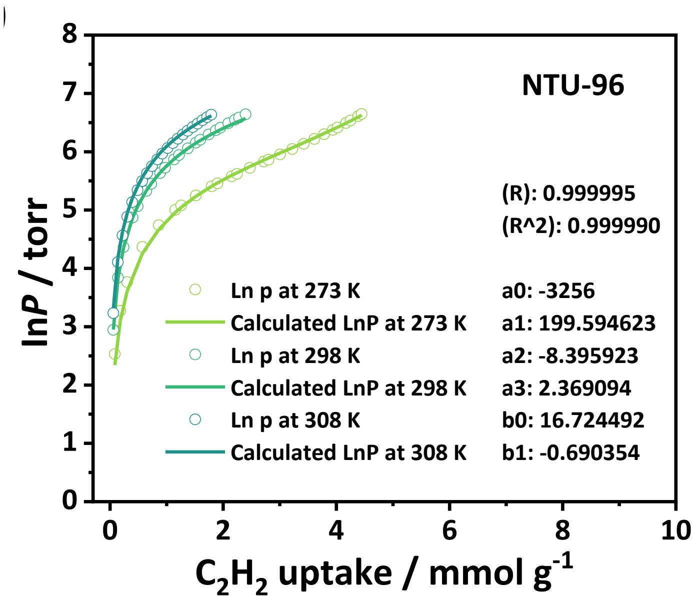

Supplementary Fig. 58. The calculated virial equation isotherms fit to the experimental C_2H_2 uptake of **NTU-96**.

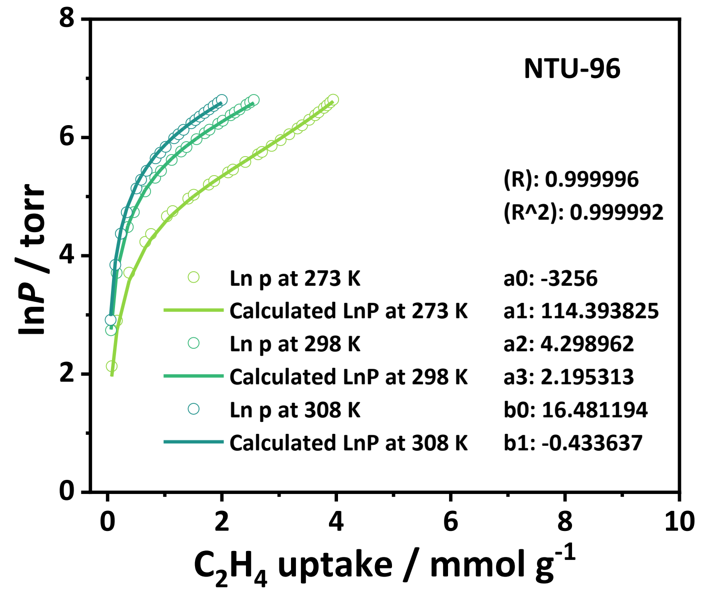

Supplementary Fig. 59. The calculated virial equation isotherms fit to the experimental C_2H_4 uptake of **NTU-96**.

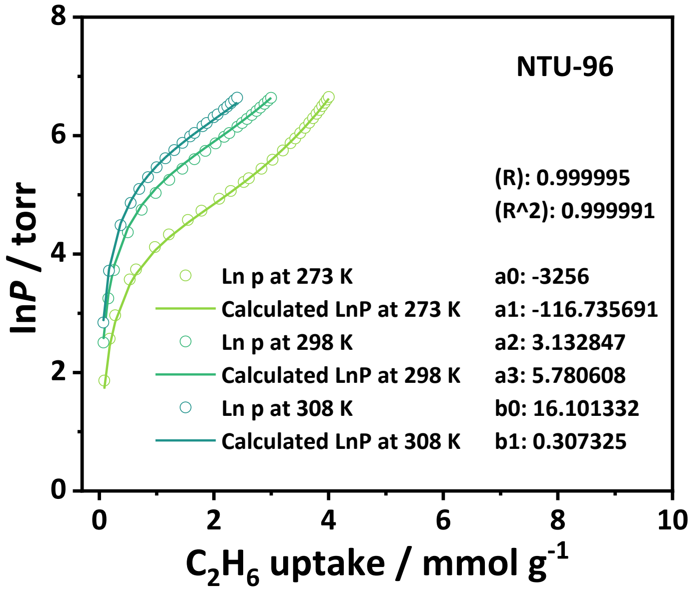

Supplementary Fig. 60. The calculated virial equation isotherms fit to the experimental C_2H_6 uptake of **NTU-96**.

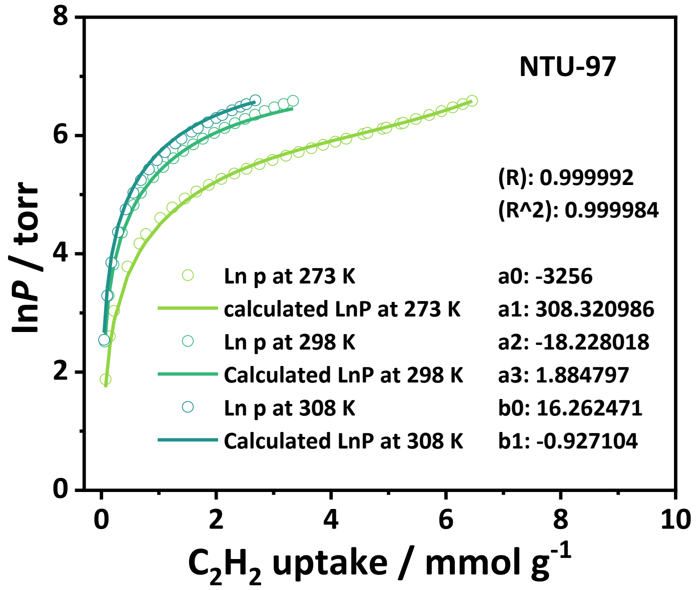

Supplementary Fig. 61. The calculated virial equation isotherms fit to the experimental C_2H_2 uptake of **NTU-97**.

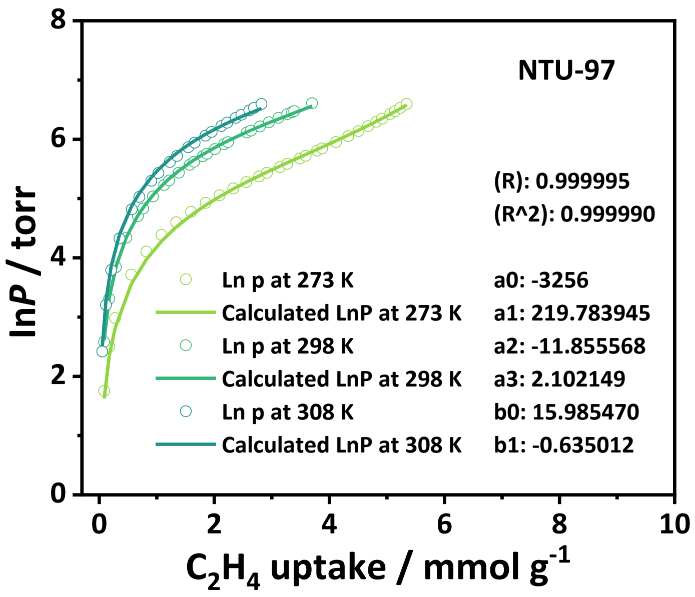

Supplementary Fig. 62. The calculated virial equation isotherms fit to the experimental C_2H_4 uptake of **NTU-97**.

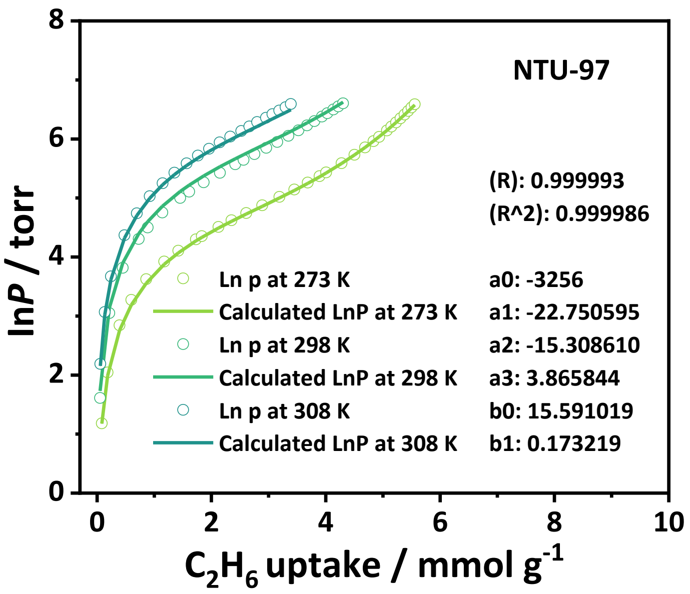

Supplementary Fig. 63. The calculated virial equation isotherms fit to the experimental C_2H_6 uptake of **NTU-97**.

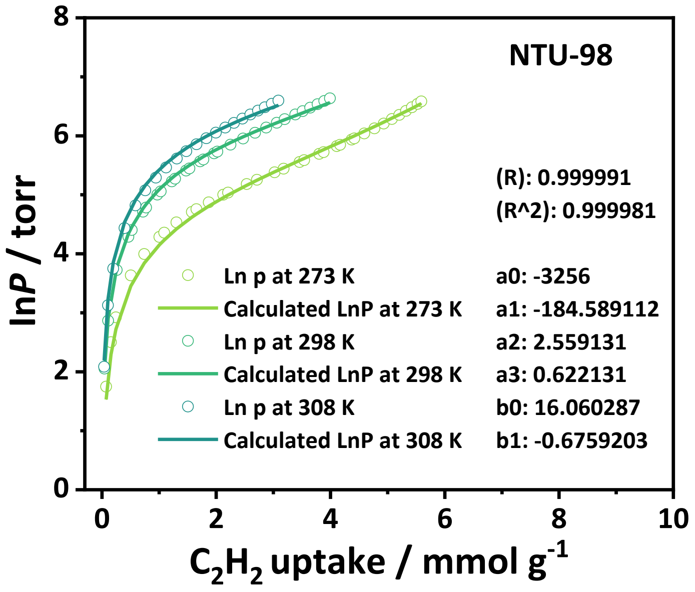

Supplementary Fig. 64. The calculated virial equation isotherms fit to the experimental C_2H_2 uptake of **NTU-98**.

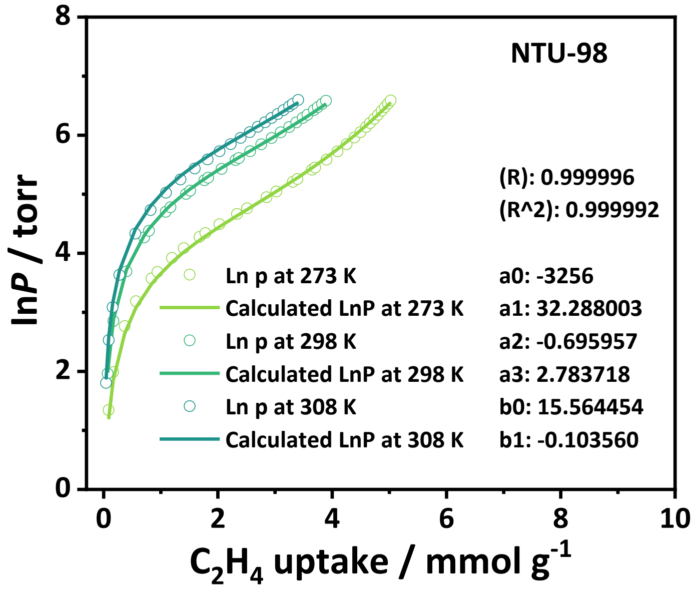

Supplementary Fig. 65. The calculated virial equation isotherms fit to the experimental C_2H_4 uptake of **NTU-98**.

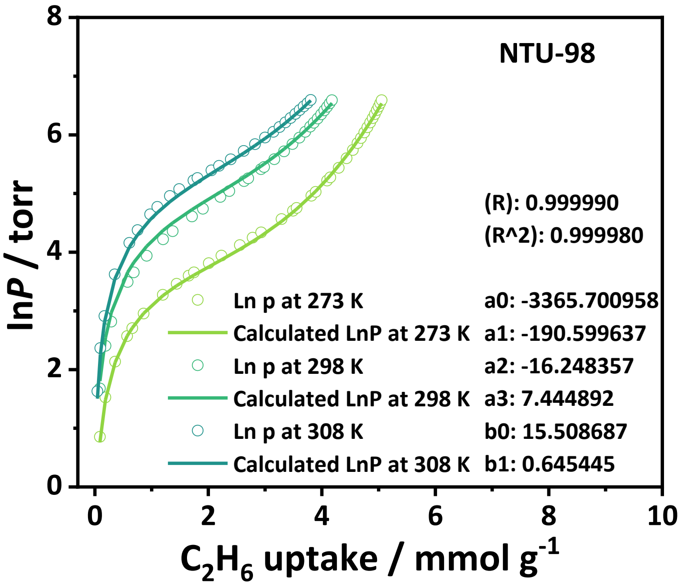

Supplementary Fig. 66. The calculated virial equation isotherms fit to the experimental C_2H_6 uptake of **NTU-98**.

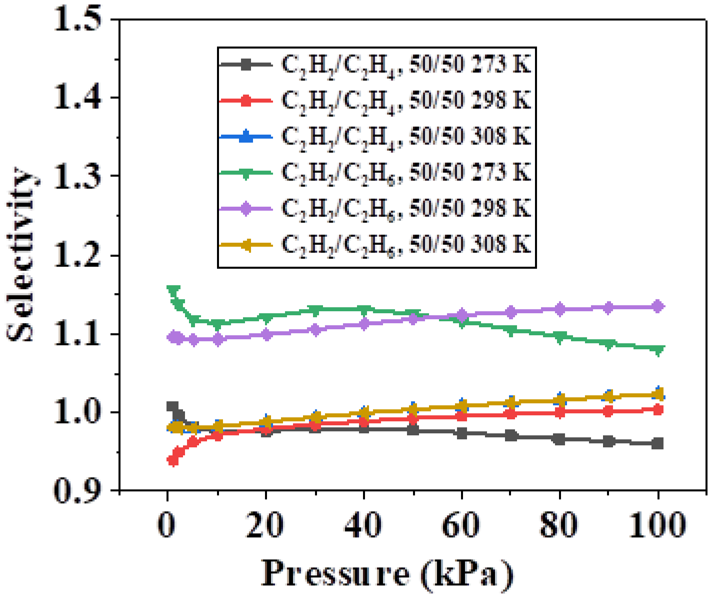

Supplementary Fig. 67. IAST selectivities of $Zn_4O(PyC)_3$ for C_2H_4/C_2H_2 (v/v:50/50) and C_2H_6/C_2H_2 (v/v:50/50) at 273 K, 298 K and 308 K.

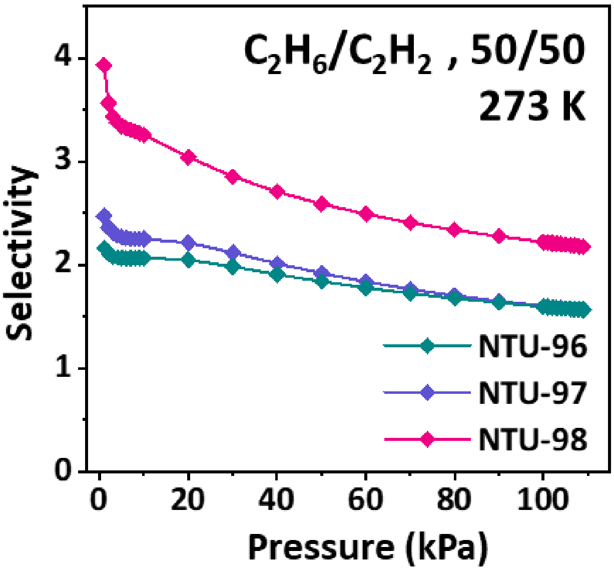

Supplementary Fig. 68. IAST selectivities of **NTU-96**, **NTU-97** and **NTU-98** for C_2H_6/C_2H_2 (v/v:50/50) at 273 K.

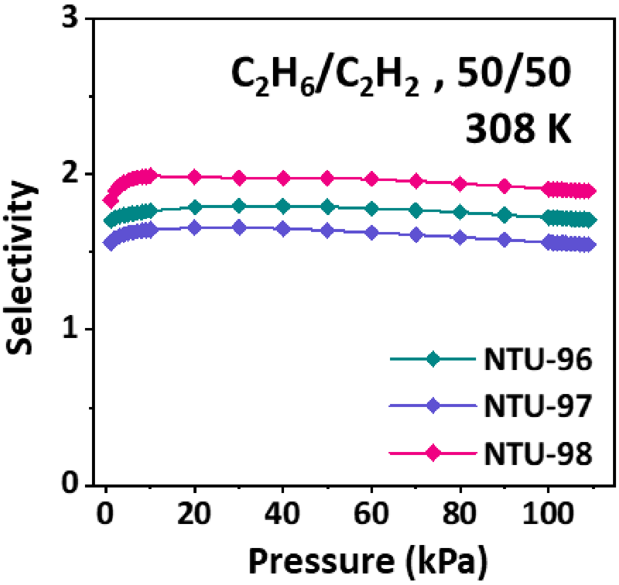

Supplementary Fig. 69. IAST selectivities of **NTU-96**, **NTU-97** and **NTU-98** for C_2H_6/C_2H_2 (v/v:50/50) at 308 K.

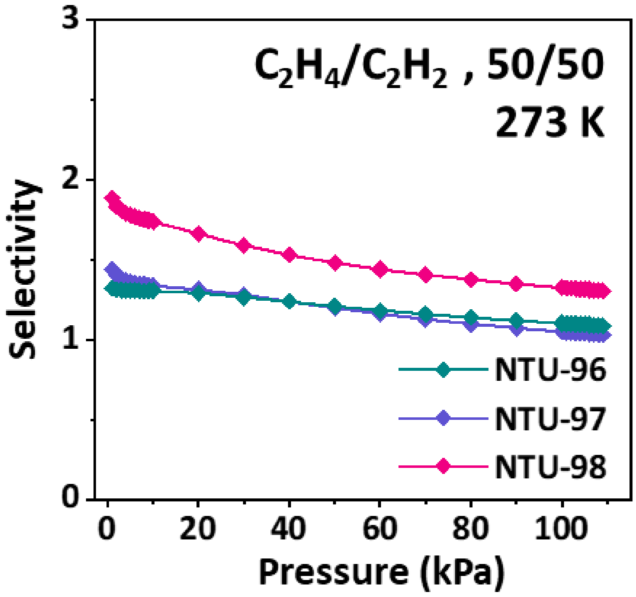

Supplementary Fig. 70. IAST selectivities of **NTU-96**, **NTU-97** and **NTU-98** for C_2H_4/C_2H_2 (v/v:50/50) at 273 K.

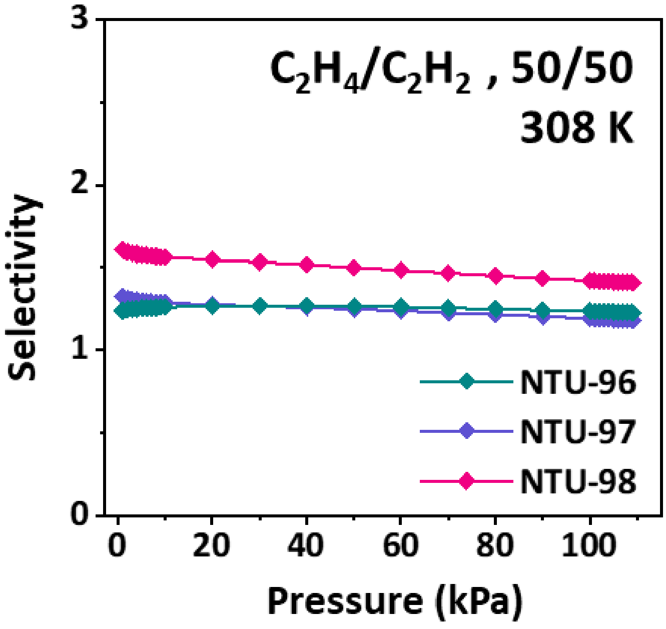

Supplementary Fig. 71. IAST selectivities of **NTU-96**, **NTU-97** and **NTU-98** for C_2H_4/C_2H_2 (v/v:50/50) at 308 K.

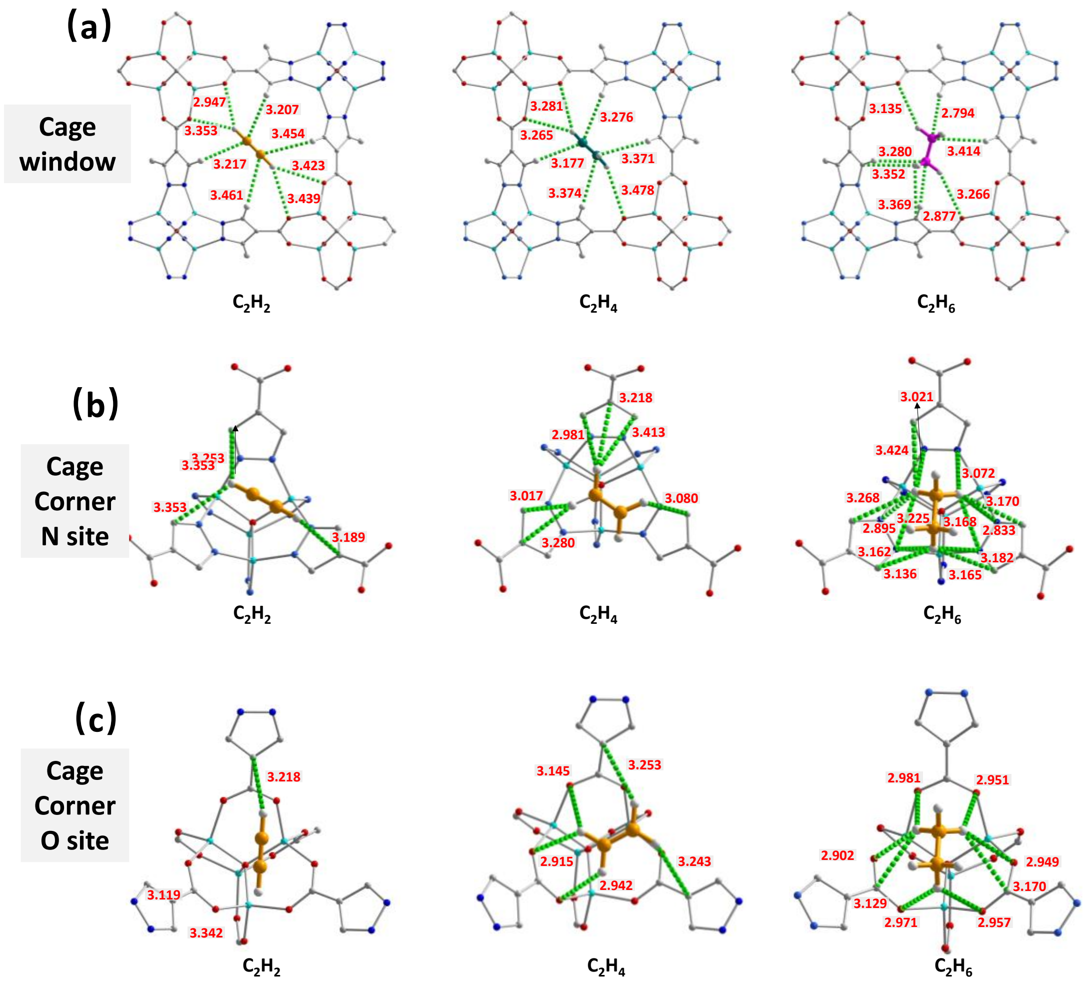

Supplementary Fig. 72. DFT calculated host-guest interactions of $Zn_4O(PyC)_3$: (a) C2 hydrocarbons inside the small cage center, (b) around cage corner N site, and (c) around cage corner O site. The green and yellow dash line indicates the formed hydrogen bonding and C-H…**π**, respectively.

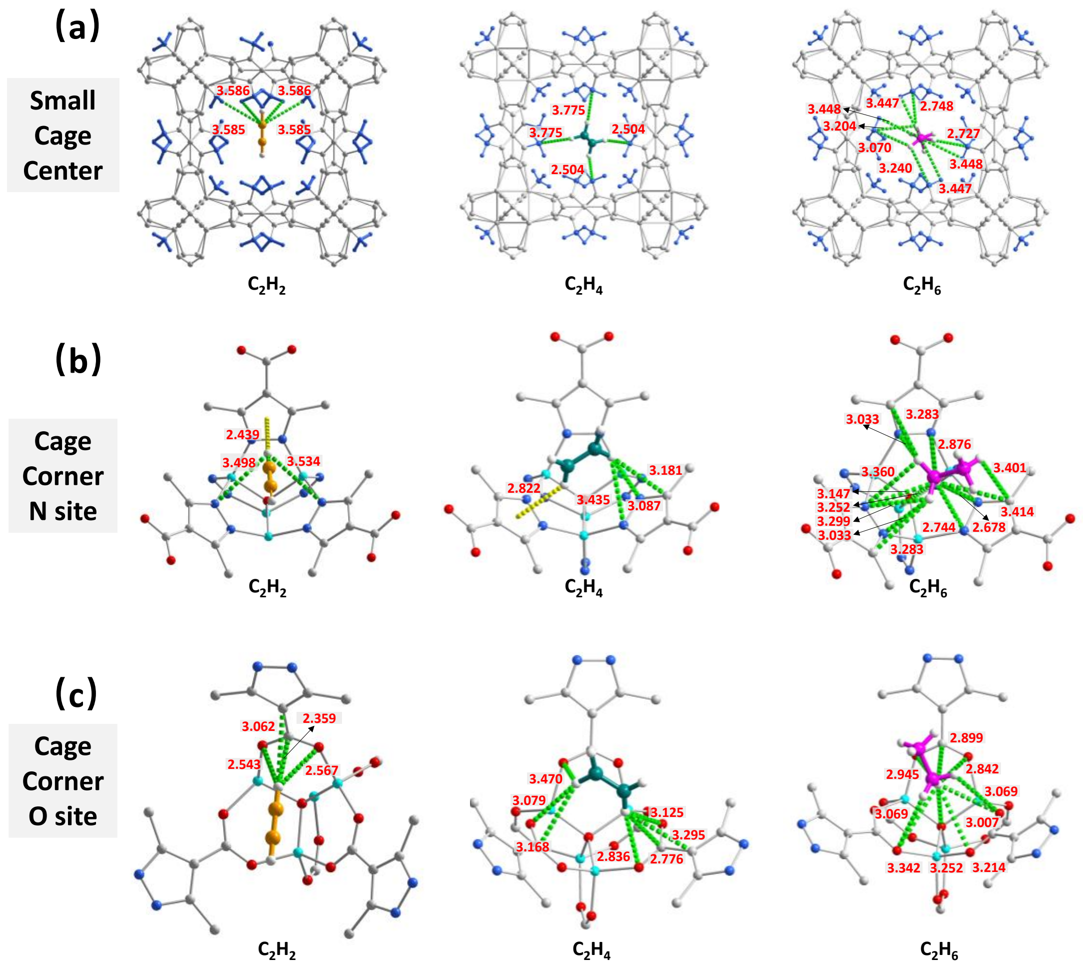

Supplementary Fig. 73. Gas-loaded structural views of **NTU-98**: (a) C2 hydrocarbons inside the small cage center, (b) around cage corner N site, and (c) around cage corner O site. The green and yellow dash line indicates the formed hydrogen bonding and C-H…**π**, respectively.

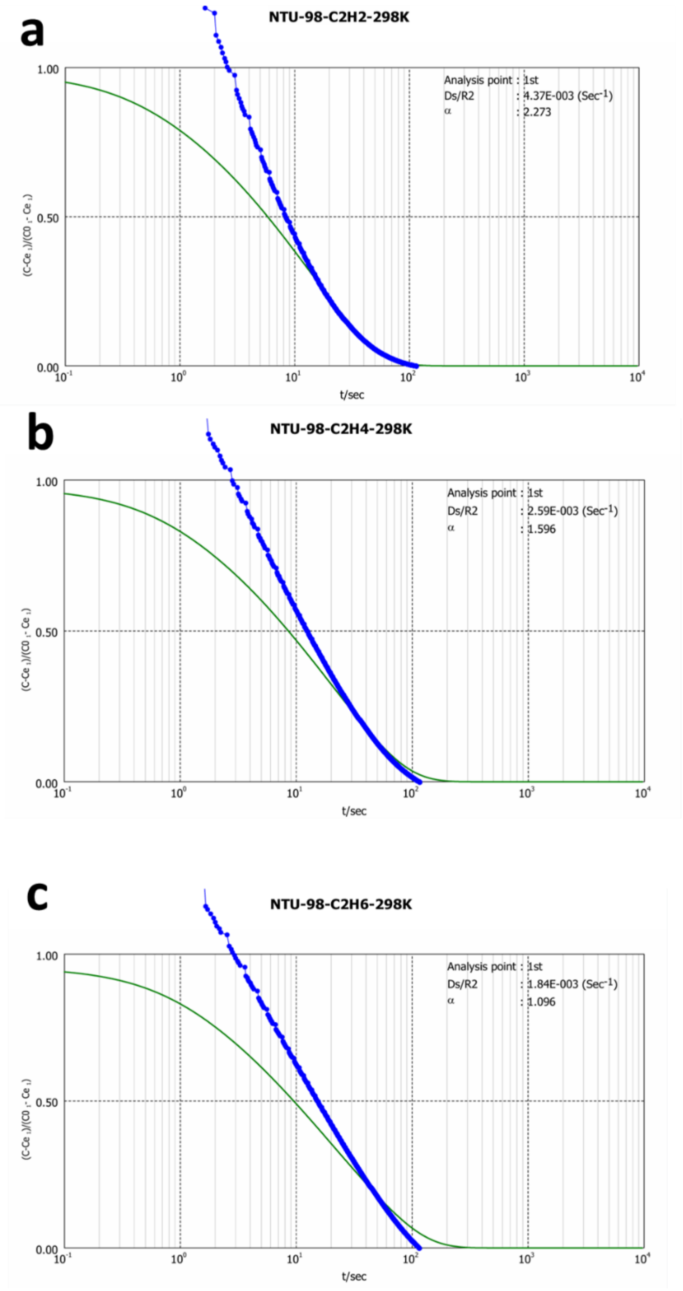

Supplementary Fig. 74. Diffusion rate of (a) C_2H_2 (b) C_2H_4 (c) C_2H_6 on **NTU-98**, fitted automatically with BEL-Dyna software according to the Crank theory. C, concentration; C^0, initial concentration; C^e, concentration at equilibrium.

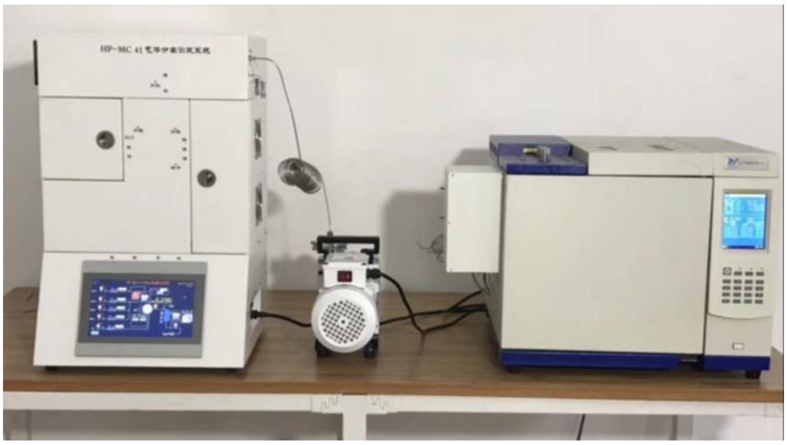

Supplementary Fig. 75. View of the breakthrough equipment (Beifang Gaorui CT-4 system).

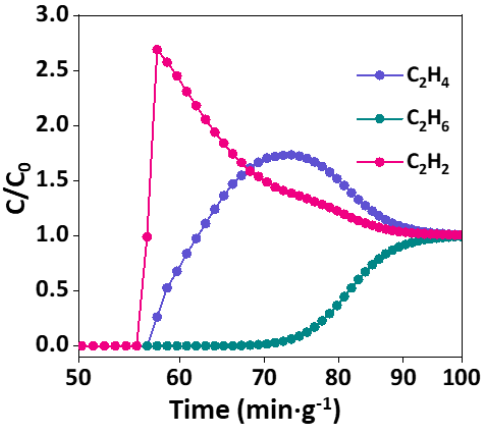

Supplementary Fig. 76. Breakthrough curves of **NTU-98** with ternary mixtures of $C_2H_2/C_2H_4/C_2H_6$ (1/1/1, v/v/v) at 298 K, 1 bar. The total flow rate is 0.9 mL·min^{-1}.

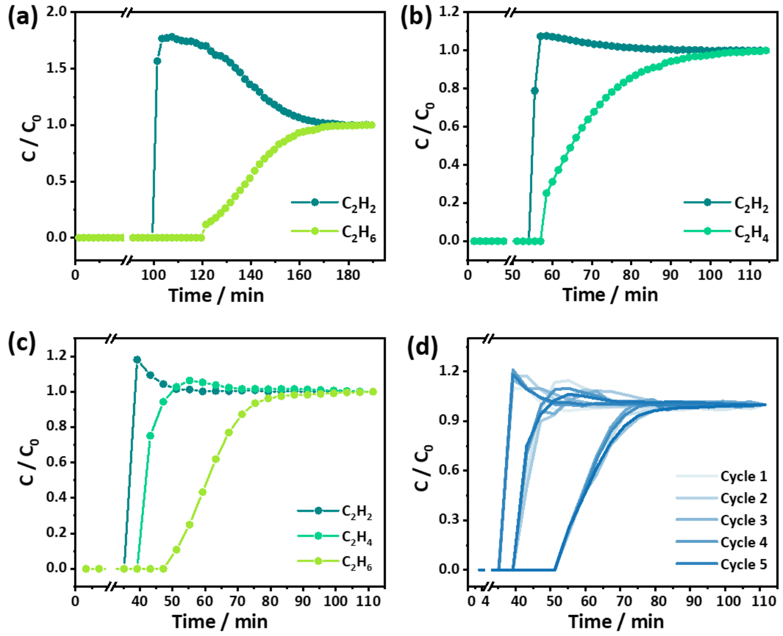

Supplementary Fig.77. Breakthrough curves of NTU-96 (2.8107 g) at 298 K, 1 bar: (a) C_2H_6/C_2H_2 mixture (1/1, v/v, 0.9 mL·min^{-1}), (b) C_2H_4/C_2H_2 mixture (1/1, v/v, 0.9 mL·min^{-1}), (c) ternary mixtures $C_2H_2/C_2H_4/C_2H_6$ (90/9/1, v/v/v, 2 mL·min^{-1}), (d) cycle breakthrough tests.

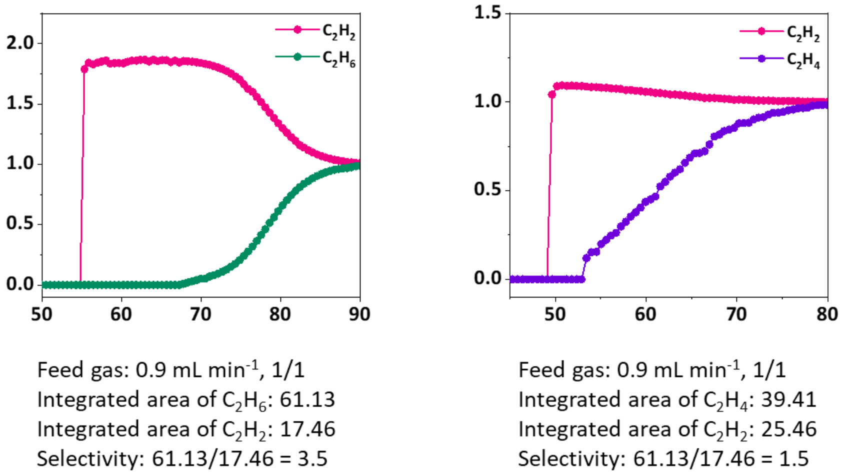

Supplementary Fig. 78. Calculated dynamic selectivities of C_2H_6/C_2H_2 and C_2H_4/C_2H_2 for **NTU-98**, based on breakthrough experiments with binary mixtures.

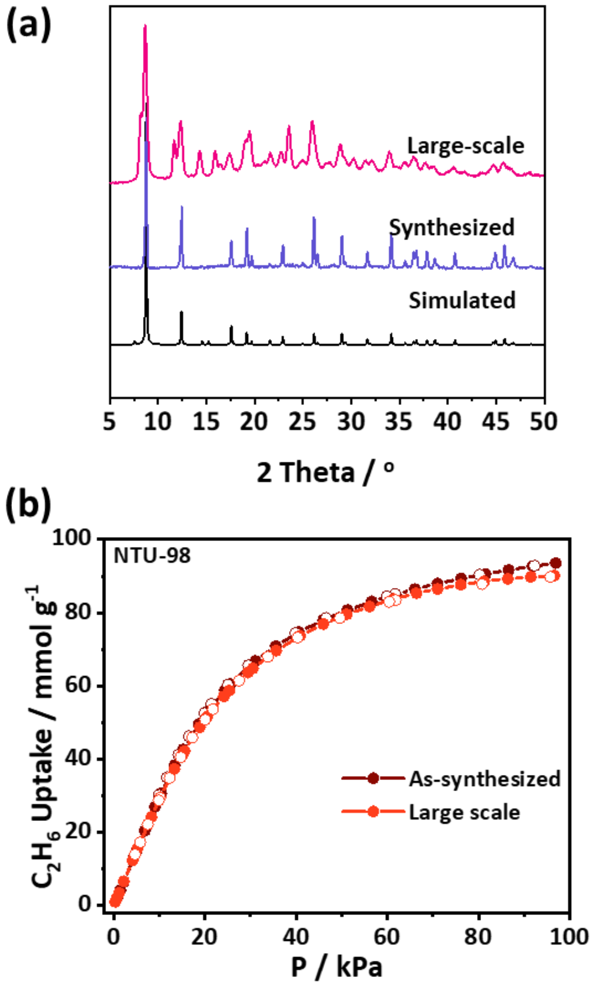

Supplementary Fig. 79. (a) PXRD of the large-scale synthesized **NTU-98** and (b) the comparison of C_2H_6 adsorption isotherms for small-scale synthesized and large-scale synthesized.

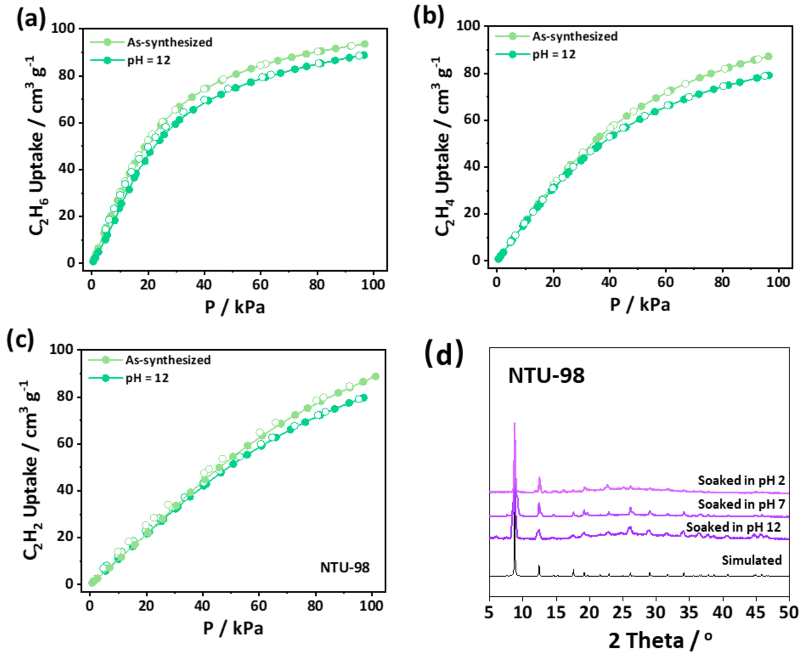

Supplementary Fig. 80. C2 hydrocarbon adsorption isotherms of **NTU-98** after chemical solution treatment for one week at 298 K (a–c), and the corresponding PXRD patterns (d).

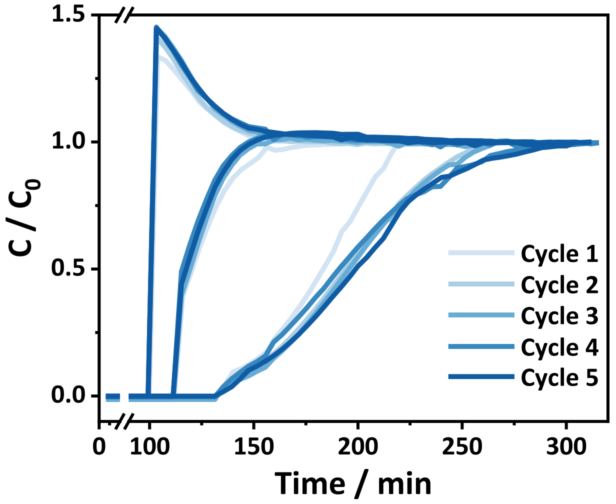

Supplementary Fig. 81. Cycling breakthrough curve of $C_2H_2/C_2H_4/C_2H_6$ (90/9/1, v/v/v) for **NTU-98** (2.771 g) at 298 K, 1 bar. The total flow rate is 2.0 mL·min^{-1}.

Supplementary Table 3. Comparison of the C2 Hydrocarbons Q_{st} and uptake difference for benchmark materials.

MOFs	T (K)	P (kPa)	C_2H_2		C_2H_4		C_2H_6		C_2H_4-C_2H_2 Uptake (cm^3/g)	C_2H_6-C_2H_2 Uptake (cm^3/g)	Ref
			Uptake (cm^3/g)	$Q_{st,n=0}$ (kJ/mol)	Uptake (cm^3/g)	$Q_{st,n=0}$ (kJ/mol)	Uptake (cm^3/g)	$Q_{st,n=0}$ (kJ/mol)			
NTU-98	298	50	53.5	23.46	64.8	27.26	80	32.16	11.3	26.5	***This work***
NTU-97	298	50	38.5	23.39	48.8	25.08	69.8	28.67	10.3	31.3	
NTU-96	298	50	26.7	26.48	33.4	27.71	47.8	29.4	6.7	21.1	
Zn-FBA	298	50	19.6	29.7	23.5	39.8	25.8	42.8	3.9	6.2	[12]
NUM-7	298	50	48.8	29.3	50.7	30	59.1	35.8	1.9	10.3	[13]
Azole-Th-1	298	50	52.3	25.4	54.1	26.1	73.5	28.6	1.8	21.2	[14]
NPU-2	298	50	50.1	20.98	43.3	18.18	63.5	19.64	-6.8	13.4	[15]
MOF-525	298	50	34.5	15.81	26.7	16.74	32.6	19.95	-7.8	-1.9	[16]
BSF-1	298	50	42.3	30.7	30	26	29.6	28.6	-12.3	-12.7	[17]
Cu-FINA-2	298	50	28.4	12.3	15	20.84	9.1	——	-13.4	-19.3	[18]
HIAM-210	298	50	51.2	34.31	35.6	22.72	44.7	31.24	-15.6	-6.5	[19]
NTU-73-CH_3	298	50	72.2	37.06	53.1	29.9	63.8	35.43	-19.1	-8.4	[20]
[Zn(BDC)(H_2BPZ)] $4H_2O$	298	50	81.7	28.7	62.5	23.2	70.9	31.8	-19.2	-10.8	[21]
TJT-100	298	50	81.3	31	58.8	25	69.3	29	-22.5	-12	[22]
Uio-67-(NH_2)$_2$	298	50	86.1	27.4	57.7	24.5	78	26.5	-28.4	-8.1	[23]
Al-PyDC	296	50	126.6	35.3	52	27.8	70.6	30.1	-74.6	-56	[24]

References

1. Sheldrick GM. A short history of SHELX. *Acta Crystallogr Sec A* **64**, 112-122 (2008).

2. Spek AL. *PLATON, A Multipurpose Crystallographic Tool (Utrecht University,2001)*.

3. Vandersluis P, Spek AL. BYPASS - An effective method for the refinement of crystal structures containing disordered solvent regions. *Acta Crystallogr Sec A* **46**, 194-201 (1990).

4. Bae YS*, et al.* Separation of CO_2 from CH_4 using mixed-ligand metal-organic frameworks. *Langmuir* **24**, 8592-8598 (2008).

5. Cessford NF, Seaton NA, Duren T. Evaluation of Ideal Adsorbed Solution Theory as a Tool for the Design of Metal-Organic Framework Materials. *Ind Eng Chem Res* **51**, 4911-4921 (2012).

6. Babarao R, Hu ZQ, Jiang JW, Chempath S, Sandler SI. Storage and separation of CO_2 and CH_4 in silicalite, C-168 schwarzite, and IRMOF-1: A comparative study from monte carlo simulation. *Langmuir* **23**, 659-666 (2007).

7. Goetz V, Pupier O, Guillot A. Carbon dioxide-methane mixture adsorption on activated carbon. *Adsorption* **12**, 55-63 (2006).

8. Giannozzi P*, et al.* Advanced capabilities for materials modelling with Quantum ESPRESSO. *J Phys: Condens Matter* **29**, 465901 (2017).

9. Grimme S, Antony J, Ehrlich S, Krieg H. A consistent and accurate ab initio parametrization of density functional dispersion correction (DFT-D) for the 94 elements H-Pu. *J Chem Phys* **132**, (2010).

10. Su K, Wang W, Du S, Ji C, Yuan D. Efficient ethylene purification by a robust ethane-trapping porous organic cage. *Nat Commun* **12**, 3703 (2021).

11. Xie X-J*, et al.* Surface engineering on a microporous metal–organic framework to boost ethane/ethylene separation under humid conditions. *Chem Sci* **14**, 11890-11895 (2023).

12. Yang L*, et al.* Adsorption in Reversed Order of C2 Hydrocarbons on an Ultramicroporous Fluorinated Metal-Organic Framework. *Angew Chem Int Ed* **61**, e202204046 (2022).

13. Sun F-Z, Yang S-Q, Krishna R, Zhang Y-H, Xia Y-P, Hu T-L. Microporous Metal–Organic Framework with a Completely Reversed Adsorption Relationship for C2 Hydrocarbons at Room Temperature. *ACS Appl Mater Interfaces* **12**, 6105-6111 (2020).

14. Xu Z*, et al.* A robust Th-azole framework for highly efficient purification of C_2H_4 from a $C_2H_4/C_2H_2/C_2H_6$ mixture. *Nat Commun* **11**, 3163 (2020).

15. Zhu B, *et al.* Pore Engineering for One-Step Ethylene Purification from a Three-Component Hydrocarbon Mixture. *J Am Chem Soc* **143**, 1485-1492 (2021).

16. Wang Y, *et al.* One-step Ethylene Purification from an Acetylene/Ethylene/Ethane Ternary Mixture by Cyclopentadiene Cobalt-Functionalized Metal–Organic Frameworks. *Angew Chem Int Ed* **60**, 11350-11358 (2021).

17. Zhang Y, Yang L, Wang L, Duttwyler S, Xing H. A Microporous Metal-Organic Framework Supramolecularly Assembled from a CuII Dodecaborate Cluster Complex for Selective Gas Separation. *Angew Chem Int Ed* **58**, 8145-8150 (2019).

18. Wu X-Q, Liu J-H, He T, Zhang P-D, Yu J, Li J-R. Understanding how pore surface fluorination influences light hydrocarbon separation in metal–organic frameworks. *Chem Eng J* **407**, 127183 (2021).

19. Liu J, Wang H, Li J. Pillar-layer Zn–triazolate–dicarboxylate frameworks with a customized pore structure for efficient ethylene purification from ethylene/ethane/acetylene ternary mixtures. *Chem Sci* **14**, 5912-5917 (2023).

20. Li Y, Wu Y, Zhao J, Duan J, Jin W. Systemic regulation of binding sites in porous coordination polymers for ethylene purification from ternary C2 hydrocarbons. *Chem Sci* **15**, 9318-9324 (2024).

21. Wang G-D, Li Y-Z, Shi W-J, Hou L, Wang Y-Y, Zhu Z. One-Step C_2H_4 Purification from Ternary $C_2H_6/C_2H_4/C_2H_2$ Mixtures by a Robust Metal ‒ Organic Framework with Customized Pore Environment. *Angew Chem Int Ed* **61**, e202205427 (2022).

22. Hao H-G, *et al.* Simultaneous Trapping of C_2H_2 and C_2H_6 from a Ternary Mixture of $C_2H_2/C_2H_4/C_2H_6$ in a Robust Metal–Organic Framework for the Purification of C_2H_4. *Angew Chem Int Ed* **57**, 16067-16071 (2018).

23. Gu X-W, *et al.* Immobilization of Lewis Basic Sites into a Stable Ethane-Selective MOF Enabling One-Step Separation of Ethylene from a Ternary Mixture. *J Am Chem Soc* **144**, 2614-2623 (2022).

24. Wu E, *et al.* Incorporation of multiple supramolecular binding sites into a robust MOF for benchmark one-step ethylene purification. *Nat Commun* **14**, 6146 (2023).
